# Supplementary material for: Large-scale characterization of sex pheromone communication systems in Drosophila
Source: Nat Commun. 2021 Jul 6;12:4165. doi: 10.1038/s41467-021-24395-z (PMC8260797; doi:10.1038/s41467-021-24395-z)
Supplement: Supplementary file 1 — Supplementary Information [file 41467_2021_24395_MOESM1_ESM.pdf]

Supplementary Figures and Chemical syntheses

Large-scale characterization of sex pheromone communication systems in *Drosophila*

Mohammed A. Khallaf [1,2†], Rongfeng Cui [3], Jerrit Weißflog [4], Maide Erdogmus [1], Aleš Svatoš [4], Hany K. M. Dweck [1,5], Dario Riccardo Valenzano [3], Bill S. Hansson [1‡], and Markus Knaden [1†‡]

[1]Department of Evolutionary Neuroethology, Max Planck Institute for Chemical Ecology, Hans-Knöll-Straße 8, D-07745, Jena, Germany.
[2]Department of Zoology and Entomology, Faculty of Science, Assiut University, Assiut, Egypt
[3]Max Planck Institute for Biology of Ageing and CECAD Research Center at University of Cologne, Joseph- Stelzmann-Str 9b and 26, D50931, Cologne, Germany.
[4]Group of Mass Spectrometry and Proteomics, Max Planck Institute for Chemical Ecology, Hans-Knöll-Straße 8, D-07745, Jena, Germany.
[5]Present address: Department of Molecular, Cellular, and Developmental Biology, Yale University, CT 06520, New Haven, USA.
[‡]These authors jointly supervised this work
[†]Correspondence: mknaden@ice.mpg.de (M.K.); mkhallaf@ice.mpg.de (M.A.K.)

Table of Contents

Supplementary Figure 1	2
Supplementary Figure 2	4
Supplementary Figure 3	5
Supplementary Figure 4	7
Chemical syntheses	9
Supplementary References	27

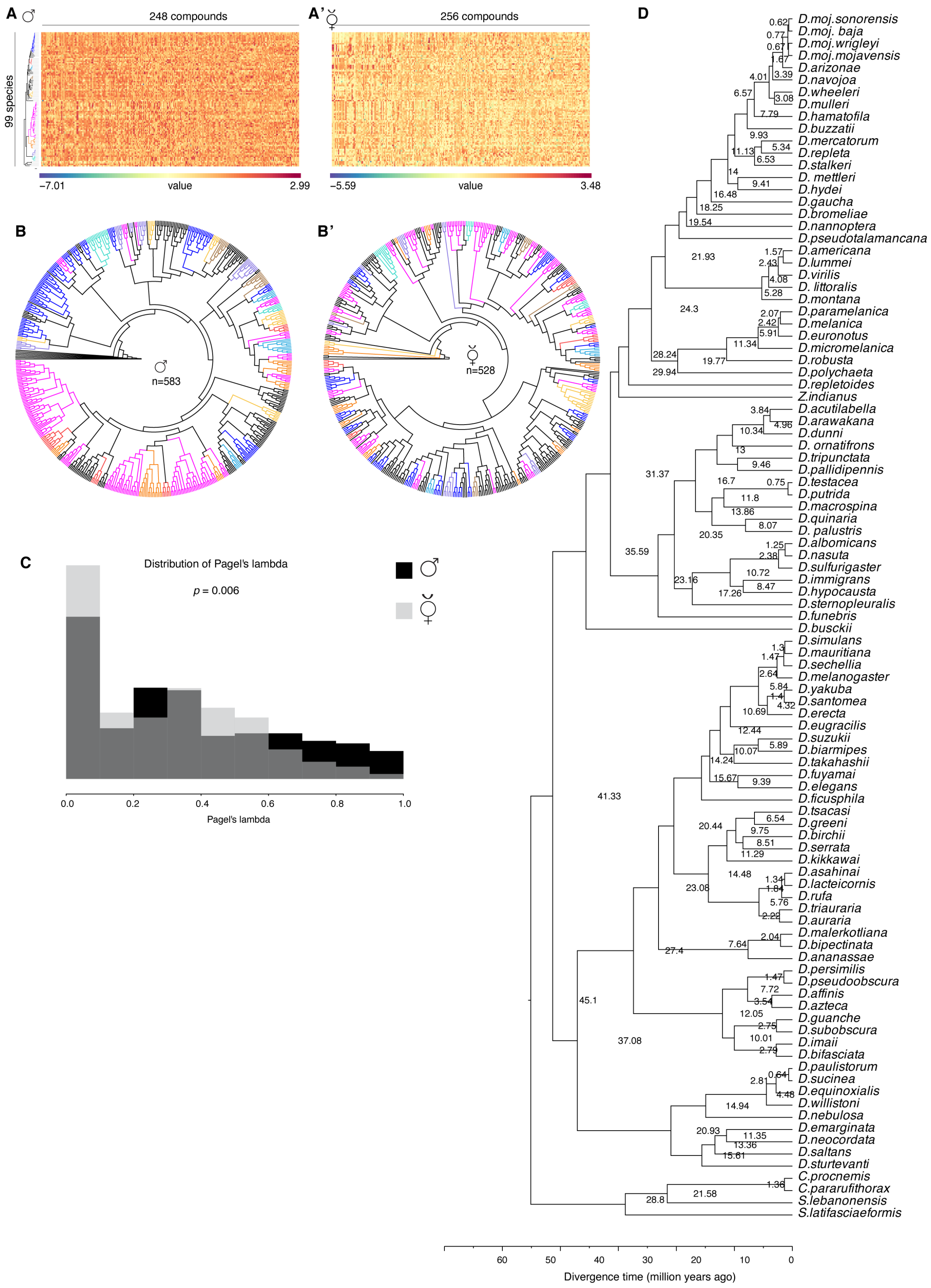

Supplementary Figure 1. Chemical analyses of 99 species within the family Drosophilidae

(A) Heat map of normalized percentages of peak areas of 248 features as detected by XCMS – a bioinformatics software designed for statistical analysis of mass spectrometry data – across the males of 99 species (See Methods for details). Rows represent the species, which are ordered on each axis according to their DNA phylogenetic relationships shown on the left side, while columns represent the 248 male chemical features present across the species sorted by lambda, highest Pagel's lambda values to the left side of the panel (i.e., most correlated with phylogeny). Each cell represents the mean of the normalized percentages of the feature's peak area in the different replicates of the same species. The feature's percentage is calculated by dividing the peak area of a particular feature by the sum of areas of all features in the replicates of the same species.
(A') Normalized percentages of peak areas of 256 female chemical features across 99 female species. Similar to Supplementary Figure 1A, rows and columns represent the species and chemical features, respectively.
(B) Chemometric hierarchical cluster analysis of 583 replicates across the 99 male species. Replicates are color coded according to their species group in Fig. 1a. Cluster analysis was performed using neighbor joining (NJ) and correlation similarity index based on peaks' quantities. *S. latifasciaeformis* was used to root the Chemometric tree.
(B') Chemometric hierarchical cluster analysis of 528 females (≥5 replicates per species) based on 256 female chemical features (see Methods). Algorithm and parameter settings are similar to Supplementary Figure 1C. Note that male chemometric tree has recovered more monophyletic groups than female chemometric tree (i.e., species groups cluster in males more than in females).
(C) Frequency histogram of Pagel's lambda estimates, showing more male chemicals (black) to be concordant with the phylogeny than in females (grey). * $P = 0.006$, Two-sided Mann Whitney U test ($n = 248$ and 256 for male and female chemical compounds, respectively).
(D) Dated phylogeny of 99 species within the family Drosophilidae inferred from 13,433,544 amino acids sites that represent 11,479 genes. Estimated divergence times are from[2,3].

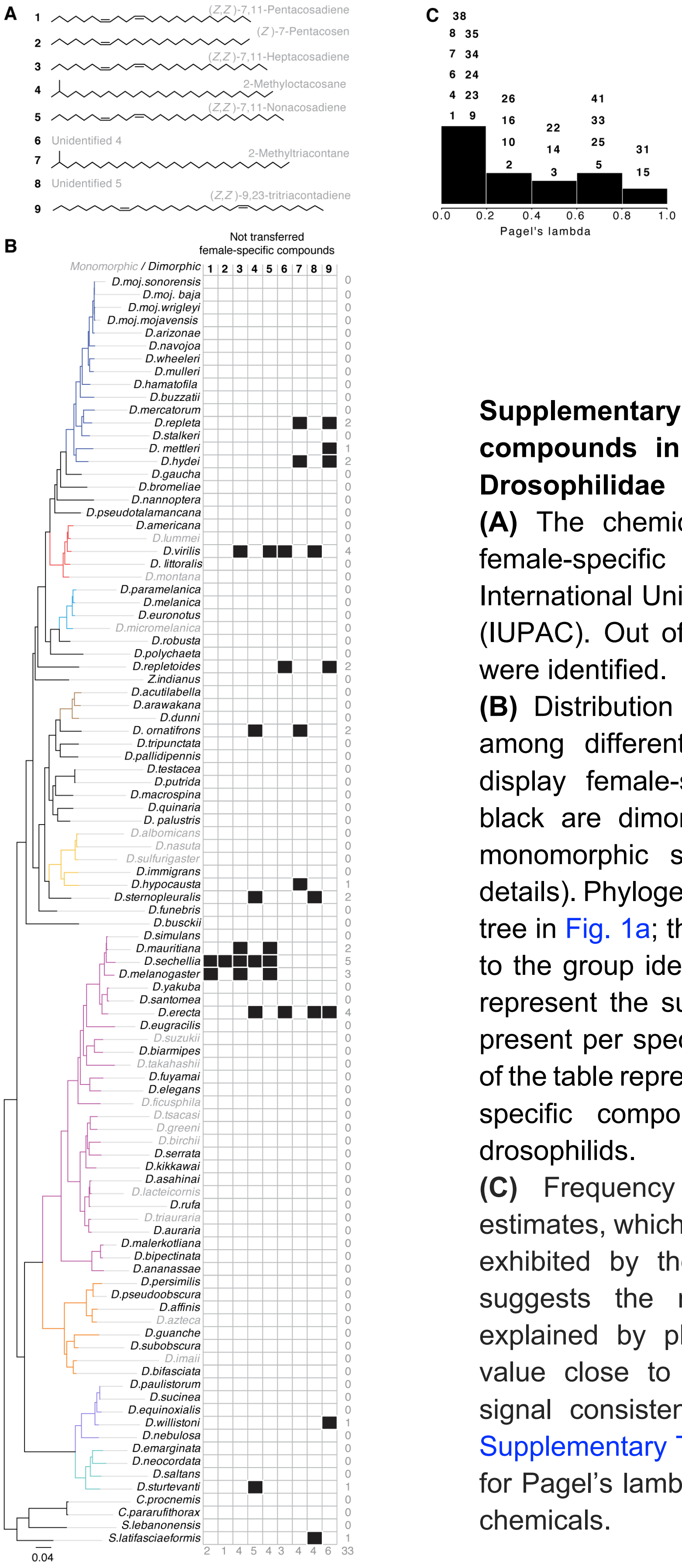

Supplementary Figure 2. The female-specific compounds in 99 species within the family Drosophilidae

(A) The chemical structure and names of the female-specific compounds according to the International Union of Pure and Applied Chemistry (IUPAC). Out of 9 female-specific compounds, 6 were identified.

(B) Distribution of 9 female-specific compounds among different drosophilids. Only 15 species display female-specific compounds. Species in black are dimorphic species, while in grey are monomorphic species (See Fig. 2b for more details). Phylogeny on the left side is identical to the tree in Fig. 1a; the branches are colored according to the group identities. Numbers on the right side represent the sum of female-specific compounds present per species, while numbers at the bottom of the table represent number of times each female-specific compound appeared in the different drosophilids.

(C) Frequency histogram of Pagel's lambda estimates, which measures the phylogenetic signal exhibited by the chemical trait[1]. A value of 0 suggests the male chemical trait cannot be explained by phylogenetic relatedness, while a value close to 1 suggests strong phylogenetic signal consistent with stabilizing selection. See Supplementary Table 2; Supplementary Figure 1C for Pagel's lambda estimates for male and female chemicals.

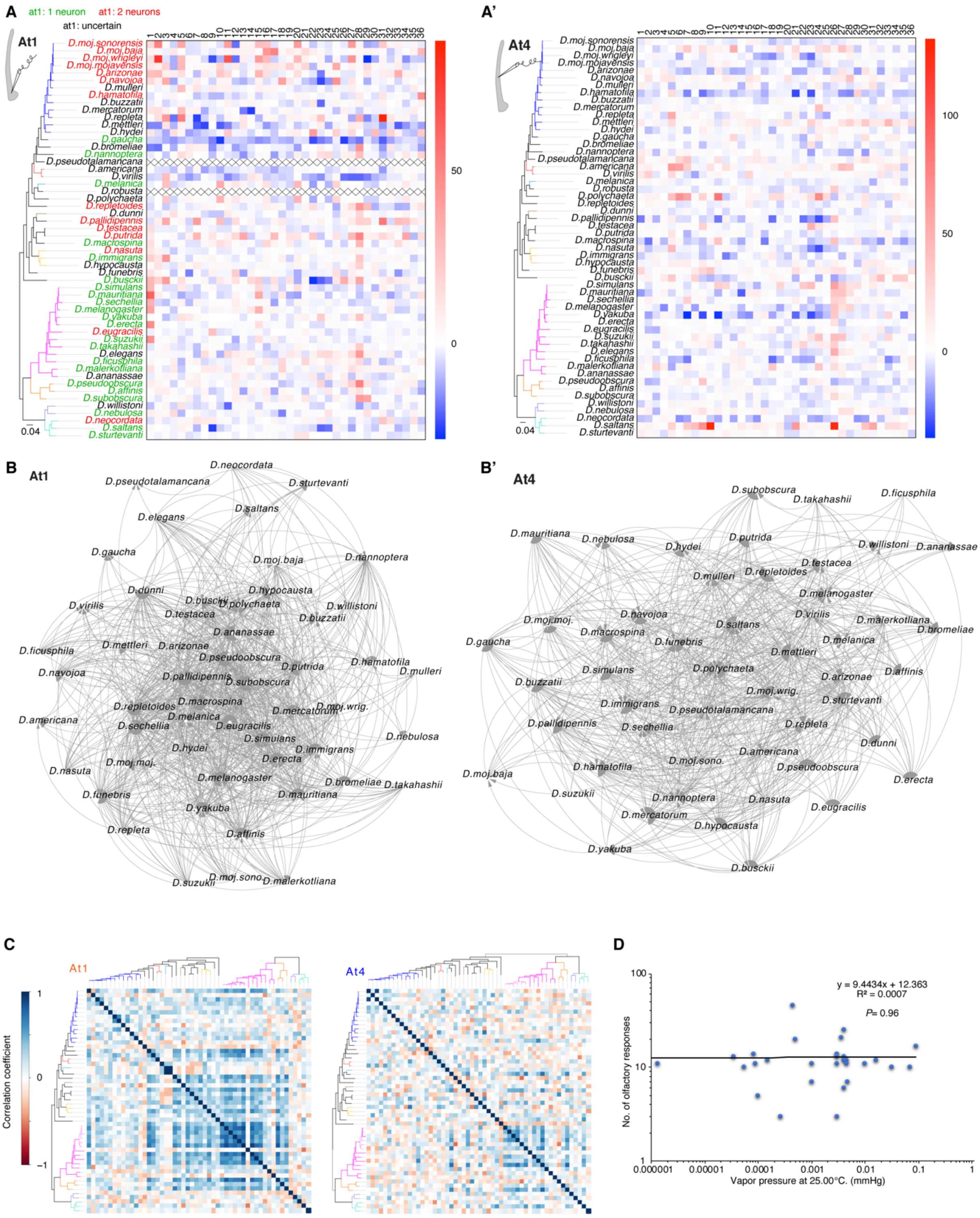

Supplementary Figure 3. The intra- and inter-specific communication through trichoid sensilla

(A) Responses of single sensillum recordings of at1 sensillum (Left: schematic drawing) in 54 species to 36 compounds used in Fig. 3b. Species names are arranged according to their phylogenetic relationship; the tree branches are colored according to the group identities. Species names are colored to reflect the presence of one (green) or two (red) neurons in at1 sensilla[4]. Due to technical difficulties, neurons in

at1 of species colored in black could not be counted by spike sorting. Note that at1 in *D. pseudotalamancana* and *D. robusta* could not be found. Color codes in the heat map illustrate the response values, which range from blue (inhibition) through white (no response) to red (activation).

(A') Electrophysiological responses of at4 neurons in 54 species to 36 compounds used in Fig. 3b.

(B) Species-species interaction network through at1 sensillum. The directed edges (i.e., arrows that connect the 53 species (i.e., nodes)) represent the olfactory interaction between species pairs (2076 pairs). Direction of the arrow points to the species which is detected. Number of species that detect itself through at1 (i.e., self-loops) is 27 out of 49. The average of correlation coefficient is 0.51 (for more details see Fig. 3d and Supplementary Table 5). The network was analyzed by Cytoscape (for more details see Methods).

(B') Species-species interaction network through at4 sensillum. Number of edges is 1559 that connect 52 species, 21 of them are able to detect their own odors by at4 (i.e., self-loops). The average of correlation coefficient is 0.292.

(C) Heat map showing the pairwise correlations between the electrophysiological responses of at1 (top) and at4 (bottom) sensilla in 54 species, which are ordered on each axis according to their phylogenetic relationships in Fig. 3b. Overall responses to 36 compounds across the 54 species were compared using Pearson correlation coefficient (R^2); Color codes in the heat map illustrate the pairwise correlations, which range from dark blue (Perfect correlation between species' responses) through white (no correlation) to dark red (perfect anticorrelation). The diagonal of the correlation matrix depicts the correlations between each species and itself (values of 1). The phylogenetic trees on each axis are similar to Fig. 3b; the branches are colored according to the group identities. Note that the male correlation matrix displays frequent dark blue cells around the diagonal and overall the matrix, i.e., high correlation coefficients only between the closely related species. Unlike the at1 correlation matrix, species' responses display lower correlation coefficients, indicating that olfactory channels in at4 evolve rapidly and independently from the phylogeny.

(D) A log-log plot of compounds' vapor pressure at 25 °C and the number of their olfactory responses in at1 and at4 of the 54 *Drosophila* species, indicating that both variables are uncorrelated.

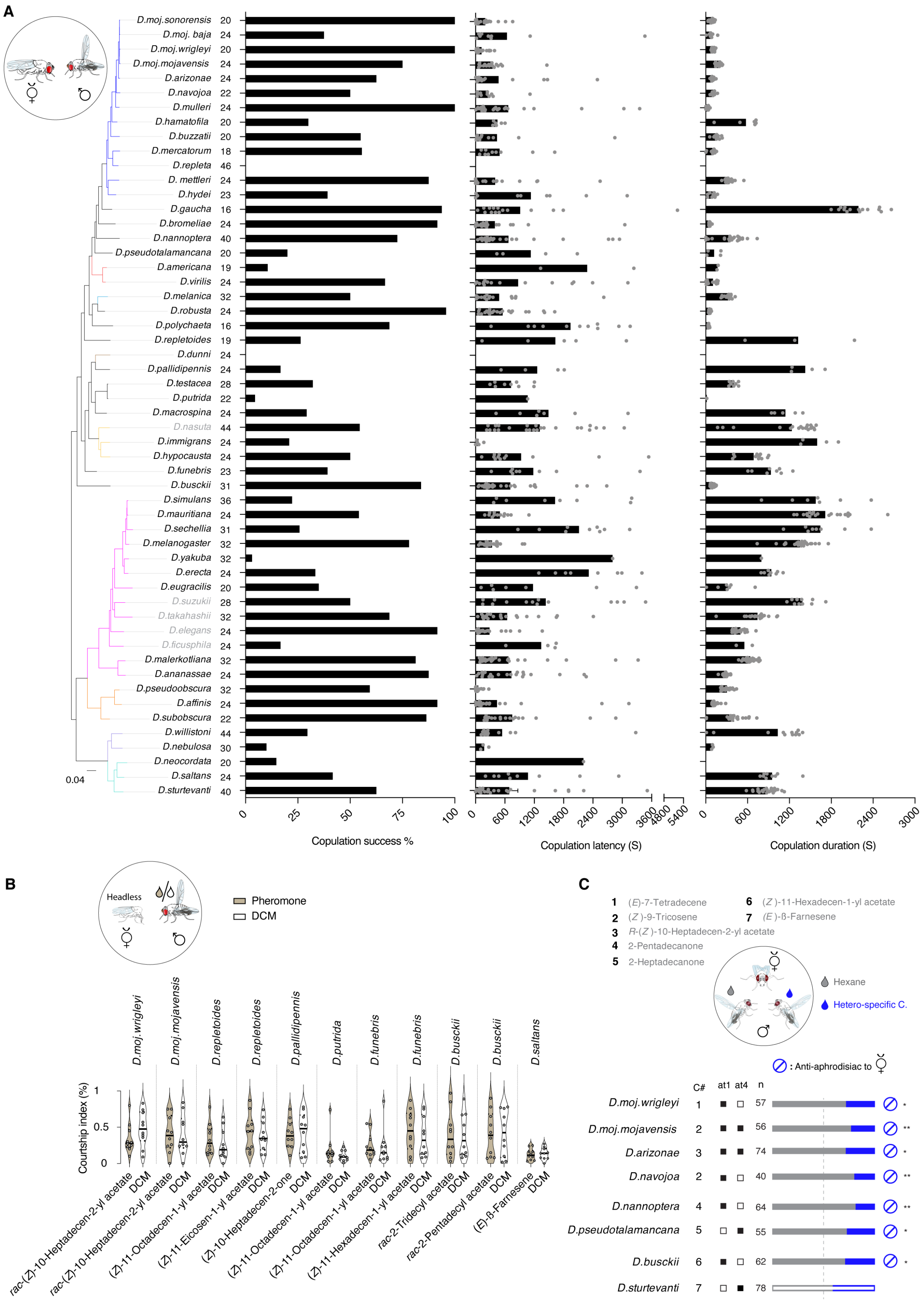

Supplementary Figure 4. Sexual behaviors of 54 species within the genus *Drosophila*

(A) Left: Copulation success [%] of virgin couples in the different species (illustrated in schematics on left side) within a 1-hour time window. Species in black are dimorphic species, while in grey are monomorphic species. For more details on the courtship behaviors of a particular species, watch its movies (Movies1 to 427, available on https://dx.doi.org/10.17617/3.5w). Species are arranged in respect to phylogeny; color of the tree branches corresponds to the species group. Number of replicates is indicated right of species names. Middle: Copulation latency in seconds (s). Males exhibiting no courtship behavior were excluded from analysis. Right: Copulation duration of the different species in seconds (s). Unlike the prolonged copulation time in *melanogaster* group (≥ 15 min)[5], copulation lasts for ~2-3 min in most species of *repleta* group. In this and the below panels, age of males and females is 10 days. Drawings made by Mohammed A. Khallaf.

(B) Left: Schematic of courtship arena where a decapitated female is courted by a conspecific male perfumed with DCM or one of the other compounds. Right, y-axis represents courtship index [%] (equal the time a male exhibits courtship behaviors / total amount of recording time (10 minutes)). Ns $P > 0.05$, Two-sided Mann Whitney U test (n = 10 replicates). Drawings made by Mohammed A. Khallaf.

(C) Top: Schematic of a mating arena where a female of each species had the choice to mate with two conspecific males perfumed with olfactory-detected heterospecific compound or solvent (hexane). Note that tested species are those that do not produce but still detect the heterospecific compound. Below: bar plots represent the percentages of copulation success of the rival males. Results from females that were only courted by one male were excluded. Drawings made by Mohammed A. Khallaf.

Chemical syntheses

Synthesis of *rac*-2-Heptylbutanoate:

2-Heptanol (1 g, 8.6 mmol) was dissolved in 20 ml CH_2Cl_2 and butyric anhydride (2.8 ml, 17.2 mmol) and triethylamine (2.4 ml, 17.2 mmol), followed by 2-3 small crystals of DMAP. The mixture was stirred at room temperature for 4 h, before water (40ml) was added. After extraction with diethylether (3 x 40 ml), the combined organic phases were washed with brine and dried over Na_2SO_4. After filtration, the solution was concentrated in vacuum and the residual oil was chromatographed on silica gel (3:1 pentane/diethylether) to obtain *rac*-2-Heptylbutanoate (1.15 g, 72% yield) as a colorless oil.
^{1}H-NMR: (400 MHz, $CDCl_3$) δ = 4.9 (sext., J = 6.3 Hz, 1H), 2.26 (t, J = 7.4 Hz, 2H), 1.65 (q, J = 7.4 Hz, 2H), 1.4-1.6 (m, 2H), 1.22-1.34 (m, 6H), 1.20 (d, J = 6.3 Hz, 3H), 0.94 (t, J = 7.4 Hz, 3H), 0.88 (t, J = 6.9 Hz, 2H) ppm; ^{13}C NMR: (100 MHz, $CDCl_3$) δ = 173.3, 70.7, 36.6, 35.9, 31.6, 25.0, 22.5, 20.0, 18.5, 13.9, 13.6 ppm.

Synthesis of (*R*) and (*S*,*Z*)-10-Heptadecen-2-yl acetate:

Synthesis of (Z)-10-Heptadecen-2-one.
Palmitoleic acid (250 mg, 0.98 mmol) was dissolved in 10 ml of THF and cooled to -78°C. A 1.6 M solution of MeLi in diethylether (1.53 ml, 2.45 mmol) was added dropwise and the mixture was allowed to warm to room temperature by removing the cooling bath. The reaction was monitored via TLC. Upon completion (after ca. 2.5h) the mixture was poured into 20 ml of 1N HCl at 0°C and extracted with diethylether (3x15 ml). The combined organic layers were washed with saturated $NaHCO_3$ (20 ml) and brine (20 ml), dried with anhydrous $NaSO_4$, filtered and concentrated in vacuo. After purification via silica gel column chromatography (12:1 hexane/EtOAc) (*Z*)-10-Heptadecen-2-one was obtained as colourless oil (225 mg, 91%).
^{1}H-NMR: (400 MHz, $CDCl_3$) δ = 5.35 (m, 2H), 2.41 (t, J = 7.3 Hz, 2H), 2.13 (s, 3H), 2.01 (q, J = 5.7 Hz, 4H), 1.57 (m, 2H), 1.29 (m, 16H), 0.89 (t, J = 6.0 Hz, 3H)ppm; ^{13}C-NMR: (100 MHz, $CDCl_3$) δ = 209.2, 130.0, 129.7, 43.8, 31.8, 29.8, 29.72, 29.68, 29.28, 29.15, 29.09, 28.97, 27.22, 27.15, 23.9, 22.6, 14.1 ppm.

Synthesis of (±,Z)-10-Heptadecen-2-yl acetate.
(*Z*)-10-Heptadecen-2-one (100 mg, 0.4 mmol) was dissolved in 20 ml of Ethanol and 15 mg $NaBH_4$ (0.4 mmol) were added. The mixture was stirred at room temperature and after 45 minutes 5ml sat. NH_4Cl solution was added. The mixture was extracted with diethylether (3x15 ml). The combined organic layers were washed with water (20 ml) and brine (20 ml), dried with anhydrous $NaSO_4$, filtered and concentrated in vacuo to obtain crude (*Z*)-10-Heptadecen-2-ol. The alcohol was dissolved in 10 ml CH_2Cl_2 and 100 µl Ac_2O, 100 µl NEt_3 and 2 crystals of DMAP were added. The mixture was stirred at room temperature overnight, quenched with 10 ml water and extracted with diethylether (3x15 ml). The combined organic layers were washed with brine (20 ml), dried with anhydrous $NaSO_4$, filtered and concentrated in vacuo. Purification via silica gel column chromatography (12:1 hexane/EtOAc) yielded racemic (*Z)-10*-Heptadecen-2-yl acetate as colourless oil (115 mg, 97%).
^{1}H-NMR: (400 MHz, $CDCl_3$) δ = 5.35 (m, 2H), 4.88 (hex, J = 6.3 Hz, 2H), 2.03 (s, 3H), 2.01 (q, J = 6.2 Hz, 4H), 1.51 (m, 2H), 1.29 (m, 18H), 1.20 (d, J = 6.4 Hz, 3H), 0.89 (t,

J = 6.8 Hz, 3H) ppm; ^{13}C-NMR: (100 MHz, $CDCl_3$) δ = 170.8, 130.0, 129.8, 71.1, 35.9, 31.8, 29.7, 29.4, 29.2, 29.0, 27.22, 27.15, 25.4, 22.6, 21.4, 19.9, 14.1 ppm; EI-MS [m/z (relative intensity)]: 41 (58), 43 (100), 54 (45), 55 (84), 67 (63), 68 (51), 69 (57), 81 (67), 82 (89), 83 (40), 95 (63), 96 (77), 97 (23), 109 (33), 110 (38), 124 (49), 138 (38), 152 (12), 166 (8), 180 (5), 194 (7), 236 (35), 281 (4 $[M-CH_3]^+$).

Separation of (R)- and (S,Z)-10-Heptadecen-2-yl acetate.
100 mg of (±,*Z*)-10-Heptadecen-2-yl acetate were dissolved in 1ml of acetone and 7 ml of phosphatebuffer (0,1M, pH = 7.0) were added to create a fine suspension. Immobilized lipase from *Candida antarctica* was added (40 mg, 2,9 U/mg) and the mixture was stirred at room temperature. The reaction was monitored via GC-MS using a chiral column (Cyclodex B, Agilent). After 20 h the mixture was extracted with diethylether (3x10 ml), washed with brine (10 ml), dried with anhydrous $NaSO_4$, filtered and concentrated in vacuo. Purification via silica gel column chromatography (9:1 hexane/EtOAc) yielded pure (*R,Z*)-10-Heptadecen-2-ol (27 mg) and (*Z*)-10-Heptadecen-2-yl acetate (67 mg of a 3:1 mixture of the (*S*)- and (*R*)-enantiomer). (*R,Z*)-10-Heptadecen-2-ol was reacetylated using the same protocol as described before to give (*R,Z*)-10-Heptadecen-2-yl acetate (25mg, ee = 99%, optical rotation: $[\alpha]^{20}_{589}$ = +1.3, 3.01 mg/ml hexane). The remaining mixture of the (*S*) and (*R*)-enantiomer was again subjected to enzymatic hydrolysis until all of the remaining (*R*)-enantiomer was hydrolysed (ca. 48h). The mixture was worked up as described before and purified via silica gel column chromatography (9:1 hexane/EtOAc) to obtain (*S,Z*)-10-Heptadecen-2-yl acetate (31 mg, ee = 97.5%, optical rotation: $[\alpha]^{20}_{589}$ = -1.4, 3.55 mg/ml hexane).

Synthesis of Heptadec-2-yl acetate:

Synthesis of Heptadecan-2-one.
Palmitic acid (1.6 g, 6.25 mmol) was dissolved in 25 ml of THF and cooled to -78°C. A 1.6 M solution of MeLi in diethylether (8.6 ml, 13.75 mmol) was added dropwise and the mixture was allowed to warm to room temperature by removing the cooling bath. The reaction was monitored via TLC. Upon completion (after ca. 2.5h) the mixture was poured into 40 ml of 1N HCl at 0°C and extracted with diethylether (3x40 ml). The combined organic layers were washed with saturated $NaHCO_3$ (50 ml) and brine (50 ml), dried with anhydrous $NaSO_4$, filtered and concentrated in vacuo. After purification via silica gel column chromatography (12:1 hexane/EtOAc) Heptadecan-2-one was obtained as white wax (1.4 g, 90%).
^{1}H-NMR: (400 MHz, $CDCl_3$) δ = 2.41 (t, J = 7.5 Hz, 2H), 2.13 (s, 3H), 1.57 (m, 2H), 1.29 (m, 26H), 0.89 (t, J = 6.8 Hz, 3H) ppm; ^{13}C-NMR: (100 MHz, $CDCl_3$) δ = 209.4, 43.8, 31.9, 29.8, 29.7, 29.7, 29.6, 29.5, 29.4, 29.3, 29.2, 23.9, 22.7, 14.1 ppm.

Synthesis of (±)-Heptadecan-2-ol.
Heptadecan-2-one (1.35 g, 5.3 mmol) was dissolved in 100 ml of ethanol and $NaBH_4$ (0.8 g, 21 mmol) was added at 0°C. The mixture was stirred for 2h at room temperature, quenched with 50 ml of sat. NH_4Cl-solution diluted with 50 ml water and extracted with diethylether (2x100 ml). The combined organic layers were washed with saturated $NaHCO_3$ (50 ml) and brine (50 ml), dried with anhydrous $NaSO_4$, filtered and concentrated in vacuo. The residue was chromatographed on silica gel (9:1 hexane/EtOAc) to yield (±)-Heptadecan-2-ol as a white wax (1.2 g, 88%).

^{1}H-NMR: (400 MHz, $CDCl_3$) δ = 3.78 (h, *J* = 5.9 Hz, 1H), 1.20-1.52 (m, 28H), 1.18 (t, *J* = 5.9 Hz, 3H), 0.88 (d, *J* = 6.6 Hz, 3H)ppm; ^{13}C-NMR: (100 MHz, $CDCl_3$) δ = 68.2, 39.4, 31.9, 29.67, 29.64, 29.61, 29.60, 29.3, 25.8, 23.5, 22.7, 14.1 ppm.

Synthesis of (±)-Heptadec-2-yl acetate.
(±)-Heptadecan-2-ol (1.17 g, 4.6 mmol) was dissolved in 20 ml CH_2Cl_2 and 940 µl Ac_2O, 1.4 ml NEt_3 and 20 mg of DMAP were added. The mixture was stirred at room temperature for 3h, quenched with 20 ml ice-water and extracted with diethylether (3x30 ml). The combined organic layers were washed with water (40 ml) and brine (40 ml), dried with anhydrous $NaSO_4$, filtered and concentrated in vacuo. Purification via silica gel column chromatography (12:1 hexane/EtOAc) yielded (±)-Heptadec-2-yl acetate as a white wax (1.36 g, 99%).
^{1}H-NMR: (400 MHz, $CDCl_3$) δ = 4.88 (h, *J* = 6.3 Hz, 1H), 2.02 (s, 3H), 1.51 (m, 2H), 1.20-1.37 (m, 26H), 1.20 (d, *J* = 6.3 Hz, 3H), 0.88 (t, *J* = 6.8 Hz, 3H)ppm; ^{13}C-NMR: (100 MHz, $CDCl_3$) δ = 170.5, 71.1, 35.9, 31.9, 29.68, 29.65, 29.61, 29.57, 29.53, 29.45, 29.35, 25.4, 22.7, 21.4, 19.9, 14.1 ppm.

Synthesis of (19*Z*,22*Z*)-Octacosadienyl acetate:

Synthesis of 3-Eicosyn-1-ol.
Freshly prepared THP-protected 3-Butynol[6] (2.5 g, 16.23 mmol) was added dropwise to a suspension of $NaNH_2$ (1.54 g of a 50% suspension in toluene, 19.5 mmol) in THF (15 ml) at 0°C. After 1h, DMSO (15 ml) and 1-Bromohexadecane (5.4 g, 17.7mmol) were added and the mixture was stirred for 3h at room temperature. The reaction mixture was quenched with water (20 ml) and extracted with diethylether (3 x 25 ml). The combined organic phases were washed with brine and dried over $MgSO_4$. After filtration, the solvent was removed via rotavap and the residue was chromatographed on silica gel (20:1 to 15:1 hexane/EtOAc) to obtain 1-[Tetrahydropyranyl)oxy]-3-eicosyne, which was immediately dissolved in 30 ml MeOH and treated with 20 mg *p*-Toluenesulfonic acid. After 1h at room temperature, 20 ml of saturated $NaHCO_3$-solution were added and the mixture was extracted with diethylether (3 x 25ml). The combined organic phases were washed with water (25 ml) and brine (15 ml) and dried over $MgSO_4$. After filtration, the solvent was removed via rotavap and the residue was chromatographed on silica gel (9:1 hexane/EtOAc) to obtain 3-Eicosyn-1-ol (2.1 g, 44% yield over two steps).
^{1}H-NMR: (400 MHz, $CDCl_3$) δ = 3.67 (t, *J* = 6.3 Hz, 2H), 2.41 (tt, ^{3}J = 9.3 Hz, ^{5}J = 2.4 Hz, 2H), 2.14 (tt, ^{3}J = 10.6 Hz, ^{5}J = 2.4 Hz, 2H), 1.48 (m, 2H), 1.30 (m, 26H), 0.89 (t, *J* = 6.8 Hz, 3H)ppm; ^{13}C-NMR: (100 MHz, $CDCl_3$) δ = 82.5, 76.3, 61.3, 31.9, 29.7, 29.6, 29.5, 29.4, 29.3, 29.2, 29.1, 29.0, 28.9, 23.1, 22.6, 18.7, 14.0 ppm.

Synthesis of 19-Eicosyn-1-ol.
Ethylene-1,2-diamine (11 ml) was cooled to 0°C and 1.02 g NaH (60% in mineral oil, 25.5 mmol) were added. The mixture was allowed to warm to room temperature and stirred for 2 h. The violet mixture was heated to 60°C and stirred for 1h, before it was cooled to 40°C. At this temperature 3-eicosyn-1-ol (1.2 g, 4 mmol) was added. The green reaction mixture was then heated to 70°C and stirred for 5 h. After cooling to 0°C HCl_{aq} (0.5 M, 20 ml) was added very carefully. The mixture was poured into a separatory funnel and HCl_{aq} (1 M, 20ml) was added before extracting with diethylether (3 x 20 ml). The combined organic phases were washed with HCl_{aq} (1 M, 30ml) and brine (30 ml), dried over Na_2SO_4 and concentrated under vacuum. Purification was

done via column chromatography on silica gel (9:1 *n*-hexane/EtOAc) and 19-Eicosyn-1-ol (780 mg, 65%) was obtained as a white waxy solid.

^{1}H-NMR: (400 MHz, $CDCl_3$) δ = 3.64 (t, J = 6.6 Hz, 2H), 2.17 (td, ^{3}J = 10.3 Hz, ^{4}J = 2.6 Hz, 2H), 1.94 (t, J = 2.7 Hz, 1H), 1.54 (m, 4H), 1.30 (m, 28H) ppm; ^{13}C-NMR: (100 MHz, $CDCl_3$) δ = 84.8, 68.0, 63.1, 32.8, 29.7, 29.6, 29.5, 29.4, 29.3, 29.1, 28.8, 28.5, 25.7, 18.4 ppm.

Synthesis of 19,22-Octacosadiyn-1-ol.

To a suspension of CuI (1.01g, 5.3 mmol), NaI (800 mg, 5.3 mmol) and K_2CO_3 (554 mg, 4 mmol) in DMF (10 ml), 1-bromo-2-octyn (550 mg, 2.91 mmol) and 19-Eicosyn-1-ol (780 mg, 2.65 mmol) were added. The suspension was stirred overnight and filtered over Celite®. The filtrate was poured into 20 ml of sat. NH_4Cl and extracted with diethylether (3 x 30 ml). The combined organic phases were washed with brine (30 ml), dried over Na_2SO_4 and concentrated under vacuum. Purification was done via column chromatography on silica gel (12:1 *n*-hexane/EtOAc) and *19*,*22*-Octacosadiyn-1-ol (737 mg, 69%) was obtained as a white waxy solid.

^{1}H-NMR: (400 MHz, $CDCl_3$) δ = 3.63 (t, J = 6.5 Hz, 2H), 3.12 (quin, ^{5}J = 2.4 Hz, 2H), 2.14 (tt, ^{3}J = 10.5 Hz, ^{5}J = 2.3 Hz, 4H), 1.52 (m, 6H), 1.30 (m, 32H), 0.90 (t, J = 6.4 Hz, 3H) ppm; ^{13}C-NMR: (100 MHz, $CDCl_3$) δ = 80.5, 74.5, 63.0, 32.8, 31.1, 29.7, 29.6, 29.5, 29.4, 29.3, 29.1, 29.0, 28.9, 28.8, 28.5, 25.7, 22.2, 18.72, 18.70, 13.9, 9.7 ppm.

Synthesis of 19,22-Octacosadiynyl acetate.

19,22-Octacosadiyn-1-ol (435 mg, 1.08 mol) were dissolved in 10 ml dichloromethane. Acetic anhydride (234 µl, 2.2 mmol), triethylamine (345 µl, 2.2 mmol) and a few crystals of 4-dimethylaminopyridine were added and the solution stirred for 2 h. Water (10 ml) was added and the mixture was extracted with diethylether (3 x 15 ml). The combined organic phases were washed with brine (30 ml), dried over Na_2SO_4 and concentrated under vacuum. Purification was done via column chromatography on silica gel (25:1 *n*-hexane/EtOAc) and 19,22-Octacosadiynyl acetate (435 mg, 90%) was obtained as a white waxy solid.

^{1}H-NMR: (400 MHz, $CDCl_3$) δ = 4.05 (t, J = 6.8 Hz, 2H), 3.11 (quin, ^{5}J = 2.4 Hz, 2H), 2.14 (tt, ^{3}J = 10.7 Hz, ^{5}J = 2.3 Hz, 4H), 2.04 (s, 3H), 1.61 (quin, J = 7.0 Hz, 2H), 1.48 (m, 4H), 1.30 (m, 32H), 0.89 (t, J = 6.7 Hz, 3H) ppm; ^{13}C-NMR: (100 MHz, $CDCl_3$) δ = 171.2, 80.4, 74.5, 64.6, 31.1, 29.7, 29.6, 29.5, 29.4, 29.3, 29.1, 28.9, 28.8, 28.6, 28.5, 25.9, 22.2, 21.0, 18.72, 18.70, 13.9, 9.7 ppm.

Synthesis of (19Z,22Z)-Octacosadienyl acetate.

19,22-Octacosadiyn-1-ol acetate (200 mg, 0.44 mmol) was dissolved in 4 ml MeOH and hydrogenated in an H_2-atmosphere in the presence of 15 mg Lindlar catalyst (Sigma). The reaction was monitored via GC-MS and after completion (ca. 15 h) the mixture was diluted with diethylether (15 ml), filtered over Celite® and concentrated in vacuum. Purification was done via reversed-phase column chromatography on C18-silica gel (3:1 MeOH/$CHCl_3$) to obtain 19*Z*,22*Z*-Octacosadienyl acetate (180 mg, 91%) as a white waxy solid.

^{1}H-NMR: (400 MHz, $CDCl_3$) δ = 5.35 (m, 4H), 4.05 (t, J = 6.8 Hz, 2H), 2.77 (t, J = 6.4 Hz, 2H), 2.05 (q, J = 6.8 Hz, 4H), 2.04 (s, 3H), 1.61 (quin, J = 7.0 Hz, 2H), 1.30 (m, 32H), 0.89 (t, J = 6.8 Hz, 3H) ppm; ^{13}C-NMR: (100 MHz, $CDCl_3$) δ = 171.2, 130.2, 128.0, 64.7, 31.5, 29.7, 29.6, 29.5, 29.4, 29.3, 29.2, 28.6, 27.25, 27.20, 25.9, 25.6, 22.6, 21.0, 14.1 ppm; EI-MS [m/z (relative intensity)]: 54 (25), 55 (100), 56 (22), 57

(41), 61 (22), 67 (50), 68 (41), 69 (78), 70 (17), 71 (18), 80 (13), 81 (53), 82 (80), 83 (71), 84 (10), 85 (10), 95 (50), 96 (78), 97 (52), 109 (31), 110 (34), 111 (22), 123 (20), 124 (21), 137 (13), 138 (14), 152 (12), 390 (37), 391 (11), 448 (17 $[M]^+$).

Synthesis of (*Z*)-11-Hexadecen-1-yl acetate:

Synthesis of Methyl (Z)-hexadec-11-enoate.

Methyl-11-bromoundecanoate (5 g, 17.9 mmol) and triphenylphosphine (4.7 g, 17.9 mmol) were dissolved in 20 ml Xylenes and refluxed overnight. The solvent was removed on the rotavap and the remaining material was dried in high vacuum overnight to obtain the crude Wittig-salt. The Wittig-salt was dissolved in 50 ml anhydrous THF and cooled to -40°C, before sodium bis(trimethylsilyl)-amide (18 ml of a 1M solution in THF, 18 mmol) was added slowly. The mixture was allowed to warm to -20°C and stirred for 30 min before it was cooled to -40°C again. Pentanal (1.03 g, 12 mmol) was added slowly and the mixture was stirred at 0°C for 1 h. The reaction mixture was diluted with hexane (50 ml), treated with water (30 ml) and saturated NH_4Cl-solution and extracted with hexane (3 x 50 ml). The combined organic phases were washed with brine and dried over $MgSO_4$. After filtration, the solvent was removed via rotavap and the residue was redissolved in hexane. The insoluble material was filtered off and washed with hexane. The filtrate was concentrated via rotavap and the residual oil was chromatographed on silica gel (20:1 to 15:1 hexane/EtOAc) to obtain methyl (*Z*)-hexadec-11-enoate (1.7 g, 53% yield) as a colorless oil.

^{1}H-NMR: (400 MHz, $CDCl_3$) δ = 5.34 (m, 2H), 3.67 (s, 3H), 2.30 (t, *J* = 7.56, 2H), 2.01 (m, 4H), 1.61 (m, 2H), 1.23-1.36 (m, 16H), 0.89 (t, *J* = 7.1 Hz, 3H) ppm; ^{13}C-NMR: (100 MHz, $CDCl_3$) δ = 174.3, 129.87, 129.85, 51.4, 34.1, 32.0, 29.7, 29.5, 29.4, 29.3, 29.2, 29.1, 27.2, 26.9, 25.0, 22.2, 14.0 ppm.

Synthesis of (Z)-hexadec-11-en-1-ol.

(*Z*)-hexadec-11-enoate (0.5 g, 1.86 mmol) was dissolved in THF/diethylether (1:1, 20ml) and cooled to 0°C. $LiAlH_4$ (1.4 g, 3.7 mmol) was added and the mixture was allowed to warm to 20°C and stirred for 30min. After cooling to 0°C, water (20 ml) was added dropwise. After extraction with diethylether (3 x 20 ml) the combined organic phases were washed with brine (30 ml), dried over $MgSO_4$ and concentrated under vacuum. Purification was done via column chromatography on silica gel (9:1 *n*-hexane/EtOAc) and (*Z*)-hexadec-11-en-1-ol (350 mg, 78%) was obtained as a colorless oil.

^{1}H-NMR: (400 MHz, $CDCl_3$) δ = 5.35 (m, 2H), 3.63 (t, *J* = 6.7 Hz, 2H), 2.01 (m, 4H), 1.56 (p, *J* = 6.9 Hz, 2H), 1.23-1.43 (m, 18H), 0.90 (t, *J* = 7.0 Hz, 3H) ppm; ^{13}C-NMR: (100 MHz, $CDCl_3$) δ = 129.87, 129.85, 63.1, 32.8, 32.0, 29.8, 29.6, 29.55, 29.5, 29.4, 29.3, 27.2, 26.9, 25.7, 22.3, 14.0 ppm.

Synthesis of (Z)-11-Hexadecen-1-yl acetate.

(*Z*)-Hexadec-11-en-1-ol (300 mg, 1.25 mmol) was dissolved in CH_2Cl_2 (10 ml) and acetic anhydride (235 µl, 2.5 mmol) and triethylamine (345 µl, 2.5 mmol) were added, followed by 2-3 small crystals of DMAP. The mixture was stirred at room temperature and monitored via TLC. After consumption of the starting material (~3h) water was added (15 ml) and the mixture extracted with diethylether (3 x 15 ml). The combined organic phases were washed with water (20 ml) and brine (20 ml), dried over Na_2SO_4 and concentrated under vacuum. The residual oil was purified via column

chromatography on silica gel (25:1 *n*-hexane/EtOAc) and (*Z*)-11-hexadecen-1-yl acetate (322 mg, 91 % yield) was obtained as a colorless oil.
^{1}H-NMR: (400 MHz, $CDCl_3$) δ = 5.35 (m, 2H), 4.05 (t, *J* = 6.8 Hz, 2H), 2.04 (s, 3H), 2.01 (m, 4H), 1.61 (p, *J* = 7.0 Hz, 2H), 1.23-1.37 (m, 18H), 0.90 (t, *J* = 7.0 Hz, 3H) ppm; ^{13}C-NMR: (100 MHz, $CDCl_3$) δ = 171.2, 129.85, 64.6, 32.0, 29.7, 29.51, 29.49, 29.3, 29.2, 28.6, 27.2, 26.9, 25.9, 22.3, 21.0, 14.0 ppm.

Synthesis of (*Z*)-10-heneicosene:

Bromooctane (1.47 g, 7.6 mmol) and triphenylphosphine (2 g, 7.6 mmol) were dissolved in 40 ml Xylene and refluxed for 24h. The solvent was removed in vacuo and the residue was dissolved in 5 ml dichloromethane. Crude phosphonium salt was precipitated by slow dilution with n-hexane as a white solid, which was collected via filtration, washed thoroughly with n-hexane to remove unreacted starting compounds and dried in vacuo.
The Wittig-salt was dissolved in 20 ml anhydrous THF and cooled to -40°C, before sodium bis(trimethylsilyl)-amide (7.4 ml of a 1M solution in THF, 7.4 mmol) was added slowly. The mixture was allowed to warm to -20°C and stirred for 30 min before it was cooled to -40°C again. A solution of tridecanal (1.23 g, 6.2 mmol) in THF (5 ml) was added slowly and the mixture allowed warming to 20°C. After stirring for 1 h, the reaction mixture was diluted with hexane (25 ml), treated with water (30 ml) and saturated NH_4Cl-solution and extracted with hexane (3 x 25 ml). The combined organic phases were washed with brine and dried over Na_2SO_4. After filtration, the solvent was removed via rotavap and the residue was redissolved in hexane. The insoluble material was filtered off and washed with hexane. The filtrate was concentrated via rotavap and the residual oil was chromatographed on silica gel (25:1 hexane/EtOAc) to obtain methyl (*Z*)-10-heneicosene (1.15 g, 63% yield) as a colorless oil.
^{1}H-NMR: (400 MHz, $CDCl_3$) δ = 5.35 (m, 2H), 2.01 (q, *J* = 6.3 Hz, 4H), 1.22-1.37 (m, 30H), 0.88 (t, *J* = 6.9 Hz, 6H) ppm; 13C NMR: (100 MHz, CDCl3) δ = 129.9, 31.9, 31.90, 29.8, 29.7, 29.64, 29.61, 29.56, 29.4, 29.3, 29.2, 27.2, 22.7, 14.1 ppm

Synthesis of (*Z*)-9-Octadecen-1-yl acetate:

Oleyl alcohol (1 g, 3.7 mmol) was dissolved in CH_2Cl_2 (40 ml) and acetic anhydride (710 μl, 7.5 mmol) and triethylamine (1.05 ml, 7.5 mmol) were added, followed by 2-3 small crystals of DMAP. The mixture was stirred at room temperature and monitored via TLC. After consumption of the starting material (~3h) water was added (25 ml) and the mixture extracted with diethylether (3 x 25 ml). The combined organic phases were washed with water (20 ml) and brine (20 ml), dried over Na_2SO_4 and concentrated under vacuum. The residual oil was purified via column chromatography on silica gel (9:1 hexane/EtOAc) and (*Z*)-9-octadecen-1-yl acetate (1.05 g, 91 % yield) was obtained as a colorless oil.
^{1}H-NMR: (400 MHz, $CDCl_3$) δ = 5.35 (m, 2H), 4.05 (t, *J* = 6.8 Hz, 2H), 2.04 (s, 3H), 2.01 (q, *J* = 6.9 Hz, 4H), 1.61 (p, *J* = 6.9 Hz, 4H), 1.22-1.38 (m, 22H), 0.88 (t, *J* = 6.7 Hz, 3H) ppm; ^{13}C-NMR: (100 MHz, CDCl3) δ = 171.2, 130.0, 129.8, 64.6, 31.9, 29.8, 29.7, 29.5, 29.4, 29.3, 29.2, 29.18, 28.6, 27.2, 27.16, 22.7, 21.0, 14.1 ppm

Synthesis of *rac*-2-Tridecyl acetate:

2-Tridecanol (1 g, 5 mmol) was dissolved in CH_2Cl_2 (40 ml) and acetic anhydride (950 µl, 10 mmol) and triethylamine (1.4 ml, 10 mmol) were added, followed by 2-3 small crystals of DMAP. The mixture was stirred at room temperature and monitored via TLC. After consumption of the starting material (~4h) water was added (25 ml) and the mixture extracted with diethylether (3 x 25 ml). The combined organic phases were washed with water (20 ml) and brine (20 ml), dried over Na_2SO_4 and concentrated under vacuum. The residual oil was purified via column chromatography on silica gel (9:1 hexane/EtOAc) and *rac*-2-tridecyl acetate (1.01 g, 84 % yield) was obtained as a colorless oil.
^{1}H-NMR: (400 MHz, $CDCl_3$) δ = 4.88 (sext., *J* = 6.3 Hz, 1H), 2.02 (s, 3H), 1.22-1.65 (m, 20H), 1.20 (d, *J* = 6.3 Hz, 3H), 0.88 (t, *J* = 6.8 Hz, 3H) ppm; ^{13}C-NMR: (100 MHz, $CDCl_3$) δ = 170.7, 71.0, 35.9, 31.9, 29.62, 29.6, 29.55, 29.5, 29.4, 29.3, 25.4, 22.7, 21.3, 19.9, 14.1 ppm

Synthesis of R- *and* S-*2-Tridecyl acetate.*
The pure enantiomers of 2-Tridecyl acetate were separated using the same protocol used for the enzymatic separation of *R*- and *S*-(Z)-10-heptadecen-2-yl acetate.

Synthesis of *rac*-2-Pentadecyl acetate:

Synthesis of rac-*2-Pentadecanol.*
2-Pentadecanone (1,4 g, 6.2 mmol) was dissolved in ethanol (100 ml) and cooled to 0°C. $NaBH_4$ (780 mg, 20.6 mmol) was added in small portions and the mixture stirred for 1h at 0°C. Saturated NH_4Cl-solution (50 ml) and water (50 ml) was added carefully, before the mixture was extracted with diethylether (3 x 80 ml). The combined organic phases were washed with water (80 ml) and brine (80 ml), dried over Na_2SO_4 and concentrated under vacuum. The residual oil was purified via column chromatography on silica gel (3:1 hexane/EtOAc) and *rac*-2-pentadecanol (1.29 g, 91% yield) was obtained as a white wax.
^{1}H-NMR: (400 MHz, $CDCl_3$) δ = 3.78 (sext., *J* = 6.0 Hz, 1H), 1.21-1.51 (m, 24H), 1.18 (d, *J* = 6.0 Hz, 3H), 0.88 (t, *J* = 6.7 Hz, 3H) ppm; ^{13}C-NMR: (100 MHz, $CDCl_3$) δ = 68.2, 39.4, 31.9, 29.7, 29.65, 29.62, 29.0, 25.7, 23.5, 22.7, 14.1 ppm.

Synthesis of rac-*2-Pentadecyl acetate.*
rac-2-Pentadecanol (1.29 g, 5.6 mmol) was dissolved in CH_2Cl_2 (40 ml) and acetic anhydride (1.06 ml, 11 mmol) and triethylamine (1.5 ml, 11 mmol) were added, followed by 2-3 small crystals of DMAP. The mixture was stirred at room temperature and monitored via TLC. After consumption of the starting material (~4h) water was added (25 ml) and the mixture extracted with diethylether (3 x 25 ml). The combined organic phases were washed with water (30 ml) and brine (30 ml), dried over $MgSO_4$ and concentrated under vacuum. The residual oil was purified via column chromatography on silica gel (9:1 hexane/EtOAc) and *rac*-2-pentadecyl acetate (1.18 g, 78% yield) was obtained as a colorless oil.
^{1}H-NMR: (400 MHz, $CDCl_3$) δ = 4.89 (sext., *J* = 6.3 Hz, 1H), 2.02 (s, 3H), 1.43-1.64 (m, 2H), 1.22-1.375 (m, 22H), 1.20 (d, *J* = 6.3 Hz, 3H), 0.88 (t, *J* = 6.8 Hz, 3H) ppm; ^{13}C-NMR: (100 MHz, $CDCl_3$) δ = 170.7, 71.0, 35.9, 31.9, 29.7, 29.62, 29.6, 29.5, 29.4, 29.3, 25.4, 22.7, 21.3, 19.9, 14.1 ppm

Synthesis of R- *and* S-*2-Pentadecyl acetate.*

The pure enantiomers of 2-Pentadecyl acetate were separated using the same protocol used for the enzymatic separation of *R*- and *S*-(Z)-10-heptadecen-2-yl acetate.

Synthesis of Farnesyl acetate:

Farnesol (1 g, 4.5 mmol) was dissolved in CH_2Cl_2 (40 ml) and acetic anhydride (850 μl, 9 mmol) and triethylamine (1.25 ml, 9 mmol) were added, followed by 2-3 small crystals of DMAP. The mixture was stirred at room temperature and monitored via TLC. After consumption of the starting material (~3h) water was added (25 ml) and the mixture extracted with diethylether (3 x 25 ml). The combined organic phases were washed with water (30 ml) and brine (30 ml), dried over $MgSO_4$ and concentrated under vacuum. The residual oil was purified via column chromatography on silica gel (18:1 hexane/EtOAc) and farnesyl acetate (1.06 g, 89% yield) was obtained as a colorless oil.
^{1}H-NMR: (400 MHz, $CDCl_3$) δ = 5.34 (t, *J* = 7.1 Hz, 1H), 5.09 (m, 4H), 4.59 (d, *J* = 7.1 Hz, 2H), 1.94-2.16 (m, 11H), 1.71 (s, 3H), 1.68 (s, 3H), 1.60 (s, 6H) ppm; ^{13}C-NMR: (100 MHz, $CDCl_3$) δ = 171.1, 142.2, 135.4, 131.3, 124.3, 123.6, 118.3, 61.4, 39.7, 39.5, 26.7, 26.2, 25.7, 21.0, 17.7, 16.4, 16.0 ppm.

Synthesis of 2-Hexyl acetate:
2-Hexyl acetate was synthesized via acetylation of 2-hexanol using the protocol of Cen *et al.*[7]. The NMR-data are in accordance with those reported in literature.

Synthesis of *rac*-3-Pentadecyl acetate:

Synthesis of rac*-3-Pentadecanol.*
A solution of 1-bromododecane (4 g, 16 mmol) in anhydrous THF (10ml) was added dropwise to a stirred suspension of magnesium shavings (400 mg, 16.5 mmol) in anhydrous THF (30 ml). After the addition was complete, the reaction temperature was kept at 40° for 1h, before the gray suspension was allowed to settle. Via a syringe 35 ml of the supernatant solution were taken up and added slowly to a stirred solution of propanal (900 mg, 15.5 mmol) in anhydrous THF (40 ml) while keeping the temperature at 20°C. After 30 min saturated NH_4Cl-solution (20 ml) and water (20 ml) were added slowly and the mixture was extracted with diethylether (3 x 50 ml). The combined organic phases were washed with brine (50 ml), dried over $MgSO_4$ and concentrated under vacuum. Purification was done via column chromatography on silica gel (15:1 to 6:1 hexane/EtOAc) and *rac*-3-pentadecanol (2.56 g, 72% yield) was obtained as a white solid.
^{1}H-NMR: (400 MHz, $CDCl_3$) δ = 3.35 (m, 1H), 1.19-1.56 (m, 24H), 0.94 (t, *J* = 7.4 Hz, 3H), 0.88 (t, *J* = 6.8 Hz, 3H) ppm; ^{13}C-NMR: (100 MHz, $CDCl_3$) δ = 73.4, 37.0, 31.9, 30.1, 29.7, 29.73, 29.6, 29.4, 25.7, 22.7, 14.1 ppm.

Synthesis of rac*-3-Pentadecyl acetate.*
rac-3-Pentadecanol (1.52 g, 6.7 mmol) was dissolved in CH_2Cl_2 (60 ml) and acetic anhydride (1.26 ml, 13.3 mmol) and triethylamine (1.85 ml, 13.3 mmol) were added, followed by 2-3 small crystals of DMAP. The mixture was stirred at room temperature and monitored via TLC. After consumption of the starting material (~3h) water was added (40 ml) and the mixture extracted with diethylether (3 x 50 ml). The combined organic phases were washed with water (50 ml) and brine (50 ml), dried over Na_2SO_4

and concentrated under vacuum. The residual oil was purified via column chromatography on silica gel (15:1 hexane/EtOAc) and *rac*-3-pentadecyl acetate (1.72 g, 95 % yield) was obtained as a colorless oil.
^{1}H-NMR: (400 MHz, $CDCl_3$) δ = 4.80 (p, *J* = 6.2 Hz, 1H), 2.04 (s, 3H), 1.53 (m, 4H), 1.20-1.35 (m, 20H), 0.88 (t, *J* = 7.3 Hz, 6H) ppm; ^{13}C-NMR: (100 MHz, $CDCl_3$) δ = 171.0, 75.6, 33.6, 31.9, 29.7, 29.64, 29.6, 29.5, 29.4, 26.9, 25.3, 22.7, 21.3, 14.1, 9.6 ppm.

Synthesis of (*Z*)-9-hexadecen-1-yl acetate:
Palmitoleyl alcohol (110 mg, 0.45 mmol) was dissolved in CH_2Cl_2 (4 ml) and acetic anhydride (85 μl, 0.9 mmol) and triethylamine (125 μl, 0.9 mmol) were added, followed by 1 small crystal of DMAP. The mixture was stirred at room temperature and monitored via TLC. After consumption of the starting material (~3h) water was added (5 ml) and the mixture extracted with diethylether (3 x 5 ml). The combined organic phases were washed with water (4 ml) and brine (4 ml), dried over Na_2SO_4 and concentrated under vacuum. The residual oil was purified via column chromatography on silica gel (9:1 *n*-hexane/EtOAc) and (*Z*)-9-hexadecen-1-yl acetate (115 mg, 90 % yield) was obtained as a colorless oil.
^{1}H-NMR: (400 MHz, $CDCl_3$) δ = 5.35 (m, 2H), 4.05 (t, *J* = 6.8 Hz, 2H), 2.04 (s, 3H), 2.01 (m, 4H), 1.62 (p, *J* = 6.9 Hz, 2H), 1.23-1.39 (m, 18H), 0.88 (t, *J* = 6.7 Hz, 3H) ppm; ^{13}C-NMR: (100 MHz, $CDCl_3$) δ = 171.2, 130.0, 129.8, 64.7, 31.8, 29.7, 29.4, 29.24, 29.2, 29.0, 28.6, 27.23, 27.18, 25.9, 22.7, 21.0, 14.1 ppm.

Synthesis of (*Z*)-11-Eicosen-1-yl acetate:

Synthesis of (Z)-Eicos-11-en-1-ol.
Methyl (*Z*)-eicos-11-enoate (1 g, 3.08 mmol) was dissolved in anhydrous diethylether (50ml) and cooled to 0°C. $LiAlH_4$ (234 mg, 6.16 mmol) was added and the mixture was allowed to warm to 20°C and stirred for 1 h. After cooling to 0°C, water (40 ml) and sat. NH_4Cl-solution (40 ml) was added dropwise. The mixture was extracted with *n*-hexane (3 x 50 ml), the combined organic phases were washed with brine (30 ml) and dried over $MgSO_4$. The solvent was removed in vacuum and the obtained material was dried. Further purification was not deemed necessary. (*Z*)-eicos-11-en-1-ol (910 mg, 99%) was obtained as a colorless wax.
^{1}H-NMR: (400 MHz, $CDCl_3$) δ = 5.35 (m, 2H), 3.63 (t, *J* = 6.7 Hz, 2H), 2.01 (q, *J* = 6.3 Hz, 4H), 1.56 (p, *J* = 7.0 Hz, 2H), 1.23-1.48 (m, 26H), 0.88 (t, *J* = 6.9 Hz, 3H) ppm; ^{13}C-NMR: (100 MHz, $CDCl_3$) δ = 129.92, 129.87, 63.1, 32.8, 31.9, 29.8, 29.6, 29.58, 29.55, 29.54, 29.4, 29.33, 29.31, 27.2, 25.8, 22.7, 14.1 ppm.

Synthesis of (Z)-11-Eicosen-1-yl acetate.
(*Z*)-Eicos-11-en-1-ol (910 mg, 3.06 mmol) was dissolved in CH_2Cl_2 (20 ml) and acetic anhydride (585 μl, 6.12mmol) and triethylamine (850 μl, 6.12 mmol) were added, followed by 2-3 small crystals of DMAP. The mixture was stirred at room temperature and monitored via TLC. After consumption of the starting material (~2h) water was added (35 ml) and the mixture extracted with *n*-hexane (3 x 40 ml). The combined organic phases were washed with water (50 ml) and brine (50 ml), dried over $MgSO_4$ and concentrated in vacuum. The residual oil was purified via column chromatography on silica gel (20:1 *n*-hexane/EtOAc) and (*Z*)-11-eicosen-1-yl acetate (940 mg, 91 % yield) was obtained as a colorless oil.

^{1}H-NMR: (400 MHz, $CDCl_3$) δ = 5.35 (m, 2H), 4.05 (t, *J* = 6.8 Hz, 2H), 2.04 (s, 3H), 2.01 (q, *J* = 6.3 Hz, 4H), 1.62 (p, *J* = 7.0 Hz, 2H), 1.20-1.38 (m, 26H), 0.88 (t, *J* = 6.9 Hz, 3H) ppm; ^{13}C-NMR: (100 MHz, $CDCl_3$) δ = 171.2, 129.92, 129.86, 64.7, 31.9, 29.8, 29.55, 29.53, 29.33, 29.3, 29.27, 28.6, 27.2, 25.9, 22.7, 21.0, 14.1 ppm.

Synthesis of *rac*-2-Heptylbutanoate:

2-Heptanol (1 g, 8.6 mmol) was dissolved in 20 ml CH_2Cl_2 and butyric anhydride (2.8 ml, 17.2 mmol) and triethylamine (2.4 ml, 17.2 mmol), followed by 2-3 small crystals of DMAP. The mixture was stirred at room temperature for 4 h, before water (40ml) was added. After extraction with diethylether (3 x 40 ml), the combined organic phases were washed with brine and dried over Na_2SO_4. After filtration, the solution was concentrated in vacuum and the residual oil was chromatographed on silica gel (3:1 pentane/diethylether) to obtain *rac*-2-Heptylbutanoate (1.15 g, 72% yield) as a colorless oil.
^{1}H-NMR: (400 MHz, $CDCl_3$) δ = 4.9 (sext., *J* = 6.3 Hz, 1H), 2.26 (t, *J* = 7.4 Hz, 2H), 1.65 (q, *J* = 7.4 Hz, 2H), 1.4-1.6 (m, 2H), 1.22-1.34 (m, 6H), 1.20 (d, *J* = 6.3 Hz, 3H), 0.94 (t, *J* = 7.4 Hz, 3H), 0.88 (t, *J* = 6.9 Hz, 2H) ppm; ^{13}C NMR: (100 MHz, $CDCl_3$) δ = 173.3, 70.7, 36.6, 35.9, 31.6, 25.0, 22.5, 20.0, 18.5, 13.9, 13.6 ppm.

Synthesis of (*R*) and (*S,Z*)-10-Heptadecen-2-yl acetate:

Synthesis of (Z)-10-Heptadecen-2-one.
Palmitoleic acid (250 mg, 0.98 mmol) was dissolved in 10 ml of THF and cooled to -78°C. A 1.6 M solution of MeLi in diethylether (1.53 ml, 2.45 mmol) was added dropwise and the mixture was allowed to warm to room temperature by removing the cooling bath. The reaction was monitored via TLC. Upon completion (after ca. 2.5h) the mixture was poured into 20 ml of 1N HCl at 0°C and extracted with diethylether (3x15 ml). The combined organic layers were washed with saturated $NaHCO_3$ (20 ml) and brine (20 ml), dried with anhydrous $NaSO_4$, filtered and concentrated in vacuo. After purification via silica gel column chromatography (12:1 hexane/EtOAc) (*Z*)-10-Heptadecen-2-one was obtained as colourless oil (225 mg, 91%).
^{1}H-NMR: (400 MHz, $CDCl_3$) δ = 5.35 (m, 2H), 2.41 (t, *J* = 7.3 Hz, 2H), 2.13 (s, 3H), 2.01 (q, *J* = 5.7 Hz, 4H), 1.57 (m, 2H), 1.29 (m, 16H), 0.89 (t, *J* = 6.0 Hz, 3H)ppm; ^{13}C-NMR: (100 MHz, $CDCl_3$) δ = 209.2, 130.0, 129.7, 43.8, 31.8, 29.8, 29.72, 29.68, 29.28, 29.15, 29.09, 28.97, 27.22, 27.15, 23.9, 22.6, 14.1 ppm.

Synthesis of (±,Z)-10-Heptadecen-2-yl acetate.
(*Z*)-10-Heptadecen-2-one (100 mg, 0.4 mmol) was dissolved in 20 ml of Ethanol and 15 mg $NaBH_4$ (0.4 mmol) were added. The mixture was stirred at room temperature and after 45 minutes 5ml sat. NH_4Cl solution was added. The mixture was extracted with diethylether (3x15 ml). The combined organic layers were washed with water (20 ml) and brine (20 ml), dried with anhydrous $NaSO_4$, filtered and concentrated in vacuo to obtain crude (*Z*)-10-Heptadecen-2-ol. The alcohol was dissolved in 10 ml CH_2Cl_2 and 100 µl Ac_2O, 100 µl NEt_3 and 2 crystals of DMAP were added. The mixture was stirred at room temperature overnight, quenched with 10 ml water and extracted with diethylether (3x15 ml). The combined organic layers were washed with brine (20 ml), dried with anhydrous $NaSO_4$, filtered and concentrated in vacuo. Purification via silica

gel column chromatography (12:1 hexane/EtOAc) yielded racemic (*Z)-10*-Heptadecen-2-yl acetate as colourless oil (115 mg, 97%).
^{1}H-NMR: (400 MHz, $CDCl_3$) δ = 5.35 (m, 2H), 4.88 (hex, *J* = 6.3 Hz, 2H), 2.03 (s, 3H), 2.01 (q, *J* = 6.2 Hz, 4H), 1.51 (m, 2H), 1.29 (m, 18H), 1.20 (d, *J* = 6.4 Hz, 3H), 0.89 (t, *J* = 6.8 Hz, 3H) ppm; ^{13}C-NMR: (100 MHz, $CDCl_3$) δ = 170.8, 130.0, 129.8, 71.1, 35.9, 31.8, 29.7, 29.4, 29.2, 29.0, 27.22, 27.15, 25.4, 22.6, 21.4, 19.9, 14.1 ppm; EI-MS [m/z (relative intensity)]: 41 (58), 43 (100), 54 (45), 55 (84), 67 (63), 68 (51), 69 (57), 81 (67), 82 (89), 83 (40), 95 (63), 96 (77), 97 (23), 109 (33), 110 (38), 124 (49), 138 (38), 152 (12), 166 (8), 180 (5), 194 (7), 236 (35), 281 (4 $[M-CH_3]^+$).

Separation of (R)- and (S,Z)-10-Heptadecen-2-yl acetate.
100 mg of (±,*Z*)-10-Heptadecen-2-yl acetate were dissolved in 1ml of acetone and 7 ml of phosphatebuffer (0,1M, pH = 7.0) were added to create a fine suspension. Immobilized lipase from *Candida antarctica* was added (40 mg, 2,9 U/mg) and the mixture was stirred at room temperature. The reaction was monitored via GC-MS using a chiral column (Cyclodex B, Agilent). After 20 h the mixture was extracted with diethylether (3x10 ml), washed with brine (10 ml), dried with anhydrous $NaSO_4$, filtered and concentrated in vacuo. Purification via silica gel column chromatography (9:1 hexane/EtOAc) yielded pure (*R,Z*)-10-Heptadecen-2-ol (27 mg) and (*Z*)-10-Heptadecen-2-yl acetate (67 mg of a 3:1 mixture of the (*S*)- and (*R*)-enantiomer). (*R,Z*)-10-Heptadecen-2-ol was reacetylated using the same protocol as described before to give (*R,Z*)-10-Heptadecen-2-yl acetate (25mg, ee = 99%, optical rotation: $[\alpha]^{20}_{589}$ = +1.3, 3.01 mg/ml hexane). The remaining mixture of the (*S*) and (*R*)-enantiomer was again subjected to enzymatic hydrolysis until all of the remaining (*R*)-enantiomer was hydrolysed (ca. 48h). The mixture was worked up as described before and purified via silica gel column chromatography (9:1 hexane/EtOAc) to obtain (*S,Z*)-10-Heptadecen-2-yl acetate (31 mg, ee = 97.5%, optical rotation: $[\alpha]^{20}_{589}$ = -1.4, 3.55 mg/ml hexane).

Synthesis of Heptadec-2-yl acetate:

Synthesis of Heptadecan-2-one.
Palmitic acid (1.6 g, 6.25 mmol) was dissolved in 25 ml of THF and cooled to -78°C. A 1.6 M solution of MeLi in diethylether (8.6 ml, 13.75 mmol) was added dropwise and the mixture was allowed to warm to room temperature by removing the cooling bath. The reaction was monitored via TLC. Upon completion (after ca. 2.5h) the mixture was poured into 40 ml of 1N HCl at 0°C and extracted with diethylether (3x40 ml). The combined organic layers were washed with saturated $NaHCO_3$ (50 ml) and brine (50 ml), dried with anhydrous $NaSO_4$, filtered and concentrated in vacuo. After purification via silica gel column chromatography (12:1 hexane/EtOAc) Heptadecan-2-one was obtained as white wax (1.4 g, 90%).
^{1}H-NMR: (400 MHz, $CDCl_3$) δ = 2.41 (t, *J* = 7.5 Hz, 2H), 2.13 (s, 3H), 1.57 (m, 2H), 1.29 (m, 26H), 0.89 (t, *J* = 6.8 Hz, 3H) ppm; ^{13}C-NMR: (100 MHz, $CDCl_3$) δ = 209.4, 43.8, 31.9, 29.8, 29.7, 29.7, 29.6, 29.5, 29.4, 29.3, 29.2, 23.9, 22.7, 14.1 ppm.

Synthesis of (±)-Heptadecan-2-ol.
Heptadecan-2-one (1.35 g, 5.3 mmol) was dissolved in 100 ml of ethanol and $NaBH_4$ (0.8 g, 21 mmol) was added at 0°C. The mixture was stirred for 2h at room temperature, quenched with 50 ml of sat. NH_4Cl-solution diluted with 50 ml water and extracted with diethylether (2x100 ml). The combined organic layers were washed with

saturated $NaHCO_3$ (50 ml) and brine (50 ml), dried with anhydrous $NaSO_4$, filtered and concentrated in vacuo. The residue was chromatographed on silica gel (9:1 hexane/EtOAc) to yield (±)-Heptadecan-2-ol as a white wax (1.2 g, 88%).
^{1}H-NMR: (400 MHz, $CDCl_3$) δ = 3.78 (h, *J* = 5.9 Hz, 1H), 1.20-1.52 (m, 28H), 1.18 (t, *J* = 5.9 Hz, 3H), 0.88 (d, *J* = 6.6 Hz, 3H)ppm; ^{13}C-NMR: (100 MHz, $CDCl_3$) δ = 68.2, 39.4, 31.9, 29.67, 29.64, 29.61, 29.60, 29.3, 25.8, 23.5, 22.7, 14.1 ppm.

Synthesis of (±)-Heptadec-2-yl acetate.
(±)-Heptadecan-2-ol (1.17 g, 4.6 mmol) was dissolved in 20 ml CH_2Cl_2 and 940 µl Ac_2O, 1.4 ml NEt_3 and 20 mg of DMAP were added. The mixture was stirred at room temperature for 3h, quenched with 20 ml ice-water and extracted with diethylether (3x30 ml). The combined organic layers were washed with water (40 ml) and brine (40 ml), dried with anhydrous $NaSO_4$, filtered and concentrated in vacuo. Purification via silica gel column chromatography (12:1 hexane/EtOAc) yielded (±)-Heptadec-2-yl acetate as a white wax (1.36 g, 99%).
^{1}H-NMR: (400 MHz, $CDCl_3$) δ = 4.88 (h, *J* = 6.3 Hz, 1H), 2.02 (s, 3H), 1.51 (m, 2H), 1.20-1.37 (m, 26H), 1.20 (d, *J* = 6.3 Hz, 3H), 0.88 (t, *J* = 6.8 Hz, 3H)ppm; ^{13}C-NMR: (100 MHz, $CDCl_3$) δ = 170.5, 71.1, 35.9, 31.9, 29.68, 29.65, 29.61, 29.57, 29.53, 29.45, 29.35, 25.4, 22.7, 21.4, 19.9, 14.1 ppm.

Synthesis of (19*Z*,22*Z*)-Octacosadienyl acetate:

Synthesis of 3-Eicosyn-1-ol.
Freshly prepared THP-protected 3-Butynol[6] (2.5 g, 16.23 mmol) was added dropwise to a suspension of $NaNH_2$ (1.54 g of a 50% suspension in toluene, 19.5 mmol) in THF (15 ml) at 0°C. After 1h, DMSO (15 ml) and 1-Bromohexadecane (5.4 g, 17.7mmol) were added and the mixture was stirred for 3h at room temperature. The reaction mixture was quenched with water (20 ml) and extracted with diethylether (3 x 25 ml). The combined organic phases were washed with brine and dried over $MgSO_4$. After filtration, the solvent was removed via rotavap and the residue was chromatographed on silica gel (20:1 to 15:1 hexane/EtOAc) to obtain 1-[Tetrahydropyranyl)oxy]-3-eicosyne, which was immediately dissolved in 30 ml MeOH and treated with 20 mg *p*-Toluenesulfonic acid. After 1h at room temperature, 20 ml of saturated $NaHCO_3$-solution were added and the mixture was extracted with diethylether (3 x 25ml). The combined organic phases were washed with water (25 ml) and brine (15 ml) and dried over $MgSO_4$. After filtration, the solvent was removed via rotavap and the residue was chromatographed on silica gel (9:1 hexane/EtOAc) to obtain 3-Eicosyn-1-ol (2.1 g, 44% yield over two steps).
^{1}H-NMR: (400 MHz, $CDCl_3$) δ = 3.67 (t, *J* = 6.3 Hz, 2H), 2.41 (tt, ^{3}J = 9.3 Hz, ^{5}J = 2.4 Hz, 2H), 2.14 (tt, ^{3}J = 10.6 Hz, ^{5}J = 2.4 Hz, 2H), 1.48 (m, 2H), 1.30 (m, 26H), 0.89 (t, *J* = 6.8 Hz, 3H)ppm; ^{13}C-NMR: (100 MHz, $CDCl_3$) δ = 82.5, 76.3, 61.3, 31.9, 29.7, 29.6, 29.5, 29.4, 29.3, 29.2, 29.1, 29.0, 28.9, 23.1, 22.6, 18.7, 14.0 ppm.

Synthesis of 19-Eicosyn-1-ol.
Ethylene-1,2-diamine (11 ml) was cooled to 0°C and 1.02 g NaH (60% in mineral oil, 25.5 mmol) were added. The mixture was allowed to warm to room temperature and stirred for 2 h. The violet mixture was heated to 60°C and stirred for 1h, before it was cooled to 40°C. At this temperature 3-eicosyn-1-ol (1.2 g, 4 mmol) was added. The green reaction mixture was then heated to 70°C and stirred for 5 h. After cooling to 0°C HCl_{aq} (0.5 M, 20 ml) was added very carefully. The mixture was poured into a

separatory funnel and HCl_{aq} (1 M, 20ml) was added before extracting with diethylether (3 x 20 ml). The combined organic phases were washed with HCl_{aq} (1 M, 30ml) and brine (30 ml), dried over Na_2SO_4 and concentrated under vacuum. Purification was done via column chromatography on silica gel (9:1 *n*-hexane/EtOAc) and 19-Eicosyn-1-ol (780 mg, 65%) was obtained as a white waxy solid.

^{1}H-NMR: (400 MHz, $CDCl_3$) δ = 3.64 (t, *J* = 6.6 Hz, 2H), 2.17 (td, ^3J = 10.3 Hz, ^4J = 2.6 Hz, 2H), 1.94 (t, *J* = 2.7 Hz, 1H), 1.54 (m, 4H), 1.30 (m, 28H) ppm; ^{13}C-NMR: (100 MHz, $CDCl_3$) δ = 84.8, 68.0, 63.1, 32.8, 29.7, 29.6, 29.5, 29.4, 29.3, 29.1, 28.8, 28.5, 25.7, 18.4 ppm.

Synthesis of 19,22-Octacosadiyn-1-ol.

To a suspension of CuI (1.01g, 5.3 mmol), NaI (800 mg, 5.3 mmol) and K_2CO_3 (554 mg, 4 mmol) in DMF (10 ml), 1-bromo-2-octyn (550 mg, 2.91 mmol) and 19-Eicosyn-1-ol (780 mg, 2.65 mmol) were added. The suspension was stirred overnight and filtered over Celite®. The filtrate was poured into 20 ml of sat. NH_4Cl and extracted with diethylether (3 x 30 ml). The combined organic phases were washed with brine (30 ml), dried over Na_2SO_4 and concentrated under vacuum. Purification was done via column chromatography on silica gel (12:1 *n*-hexane/EtOAc) and *19,22*-Octacosadiyn-1-ol (737 mg, 69%) was obtained as a white waxy solid.

^{1}H-NMR: (400 MHz, $CDCl_3$) δ = 3.63 (t, *J* = 6.5 Hz, 2H), 3.12 (quin, ^5J = 2.4 Hz, 2H), 2.14 (tt, ^3J = 10.5 Hz, ^5J = 2.3 Hz, 4H), 1.52 (m, 6H), 1.30 (m, 32H), 0.90 (t, *J* = 6.4 Hz, 3H) ppm; ^{13}C-NMR: (100 MHz, $CDCl_3$) δ = 80.5, 74.5, 63.0, 32.8, 31.1, 29.7, 29.6, 29.5, 29.4, 29.3, 29.1, 29.0, 28.9, 28.8, 28.5, 25.7, 22.2, 18.72, 18.70, 13.9, 9.7 ppm.

Synthesis of 19,22-Octacosadiynyl acetate.

19,22-Octacosadiyn-1-ol (435 mg, 1.08 mol) were dissolved in 10 ml dichloromethane. Acetic anhydride (234 μl, 2.2 mmol), triethylamine (345 μl, 2.2 mmol) and a few crystals of 4-dimethylaminopyridine were added and the solution stirred for 2 h. Water (10 ml) was added and the mixture was extracted with diethylether (3 x 15 ml). The combined organic phases were washed with brine (30 ml), dried over Na_2SO_4 and concentrated under vacuum. Purification was done via column chromatography on silica gel (25:1 *n*-hexane/EtOAc) and 19,22-Octacosadiynyl acetate (435 mg, 90%) was obtained as a white waxy solid.

^{1}H-NMR: (400 MHz, $CDCl_3$) δ = 4.05 (t, *J* = 6.8 Hz, 2H), 3.11 (quin, ^5J = 2.4 Hz, 2H), 2.14 (tt, ^3J = 10.7 Hz, ^5J = 2.3 Hz, 4H), 2.04 (s, 3H), 1.61 (quin, *J* = 7.0 Hz, 2H), 1.48 (m, 4H), 1.30 (m, 32H), 0.89 (t, *J* = 6.7 Hz, 3H) ppm; ^{13}C-NMR: (100 MHz, $CDCl_3$) δ = 171.2, 80.4, 74.5, 64.6, 31.1, 29.7, 29.6, 29.5, 29.4, 29.3, 29.1, 28.9, 28.8, 28.6, 28.5, 25.9, 22.2, 21.0, 18.72, 18.70, 13.9, 9.7 ppm.

Synthesis of (19Z,22Z)-Octacosadienyl acetate.

19,22-Octacosadiyn-1-ol acetate (200 mg, 0.44 mmol) was dissolved in 4 ml MeOH and hydrogenated in an H_2-atmosphere in the presence of 15 mg Lindlar catalyst (Sigma). The reaction was monitored via GC-MS and after completion (ca. 15 h) the mixture was diluted with diethylether (15 ml), filtered over Celite® and concentrated in vacuum. Purification was done via reversed-phase column chromatography on C18-silica gel (3:1 MeOH/$CHCl_3$) to obtain 19*Z*,22*Z*-Octacosadienyl acetate (180 mg, 91%) as a white waxy solid.

^{1}H-NMR: (400 MHz, $CDCl_3$) δ = 5.35 (m, 4H), 4.05 (t, *J* = 6.8 Hz, 2H), 2.77 (t, *J* = 6.4 Hz, 2H), 2.05 (q, *J* = 6.8 Hz, 4H), 2.04 (s, 3H), 1.61 (quin, *J* = 7.0 Hz, 2H), 1.30 (m,

32H), 0.89 (t, J = 6.8 Hz, 3H) ppm; ^{13}C-NMR: (100 MHz, $CDCl_3$) δ = 171.2, 130.2, 128.0, 64.7, 31.5, 29.7, 29.6, 29.5, 29.4, 29.3, 29.2, 28.6, 27.25, 27.20, 25.9, 25.6, 22.6, 21.0, 14.1 ppm; EI-MS [m/z (relative intensity)]: 54 (25), 55 (100), 56 (22), 57 (41), 61 (22), 67 (50), 68 (41), 69 (78), 70 (17), 71 (18), 80 (13), 81 (53), 82 (80), 83 (71), 84 (10), 85 (10), 95 (50), 96 (78), 97 (52), 109 (31), 110 (34), 111 (22), 123 (20), 124 (21), 137 (13), 138 (14), 152 (12), 390 (37), 391 (11), 448 (17 $[M]^+$).

Synthesis of (*Z*)-11-Hexadecen-1-yl acetate:

Synthesis of Methyl (Z)-hexadec-11-enoate.

Methyl-11-bromoundecanoate (5 g, 17.9 mmol) and triphenylphosphine (4.7 g, 17.9 mmol) were dissolved in 20 ml Xylenes and refluxed overnight. The solvent was removed on the rotavap and the remaining material was dried in high vacuum overnight to obtain the crude Wittig-salt. The Wittig-salt was dissolved in 50 ml anhydrous THF and cooled to -40°C, before sodium bis(trimethylsilyl)-amide (18 ml of a 1M solution in THF, 18 mmol) was added slowly. The mixture was allowed to warm to -20°C and stirred for 30 min before it was cooled to -40°C again. Pentanal (1.03 g, 12 mmol) was added slowly and the mixture was stirred at 0°C for 1 h. The reaction mixture was diluted with hexane (50 ml), treated with water (30 ml) and saturated NH_4Cl-solution and extracted with hexane (3 x 50 ml). The combined organic phases were washed with brine and dried over $MgSO_4$. After filtration, the solvent was removed via rotavap and the residue was redissolved in hexane. The insoluble material was filtered off and washed with hexane. The filtrate was concentrated via rotavap and the residual oil was chromatographed on silica gel (20:1 to 15:1 hexane/EtOAc) to obtain methyl (*Z*)-hexadec-11-enoate (1.7 g, 53% yield) as a colorless oil.

^{1}H-NMR: (400 MHz, $CDCl_3$) δ = 5.34 (m, 2H), 3.67 (s, 3H), 2.30 (t, J = 7.56, 2H), 2.01 (m, 4H), 1.61 (m, 2H), 1.23-1.36 (m, 16H), 0.89 (t, J = 7.1 Hz, 3H) ppm; ^{13}C-NMR: (100 MHz, $CDCl_3$) δ = 174.3, 129.87, 129.85, 51.4, 34.1, 32.0, 29.7, 29.5, 29.4, 29.3, 29.2, 29.1, 27.2, 26.9, 25.0, 22.2, 14.0 ppm.

Synthesis of (Z)-hexadec-11-en-1-ol.

(*Z*)-hexadec-11-enoate (0.5 g, 1.86 mmol) was dissolved in THF/diethylether (1:1, 20ml) and cooled to 0°C. $LiAlH_4$ (1.4 g, 3.7 mmol) was added and the mixture was allowed to warm to 20°C and stirred for 30min. After cooling to 0°C, water (20 ml) was added dropwise. After extraction with diethylether (3 x 20 ml) the combined organic phases were washed with brine (30 ml), dried over $MgSO_4$ and concentrated under vacuum. Purification was done via column chromatography on silica gel (9:1 *n*-hexane/EtOAc) and (*Z*)-hexadec-11-en-1-ol (350 mg, 78%) was obtained as a colorless oil.

^{1}H-NMR: (400 MHz, $CDCl_3$) δ = 5.35 (m, 2H), 3.63 (t, J = 6.7 Hz, 2H), 2.01 (m, 4H), 1.56 (p, J = 6.9 Hz, 2H), 1.23-1.43 (m, 18H), 0.90 (t, J = 7.0 Hz, 3H) ppm; ^{13}C-NMR: (100 MHz, $CDCl_3$) δ = 129.87, 129.85, 63.1, 32.8, 32.0, 29.8, 29.6, 29.55, 29.5, 29.4, 29.3, 27.2, 26.9, 25.7, 22.3, 14.0 ppm.

Synthesis of (Z)-11-Hexadecen-1-yl acetate.

(*Z*)-Hexadec-11-en-1-ol (300 mg, 1.25 mmol) was dissolved in CH_2Cl_2 (10 ml) and acetic anhydride (235 µl, 2.5 mmol) and triethylamine (345 µl, 2.5 mmol) were added, followed by 2-3 small crystals of DMAP. The mixture was stirred at room temperature and monitored via TLC. After consumption of the starting material (~3h) water was

added (15 ml) and the mixture extracted with diethylether (3 x 15 ml). The combined organic phases were washed with water (20 ml) and brine (20 ml), dried over Na_2SO_4 and concentrated under vacuum. The residual oil was purified via column chromatography on silica gel (25:1 *n*-hexane/EtOAc) and (*Z*)-11-hexadecen-1-yl acetate (322 mg, 91 % yield) was obtained as a colorless oil.
^{1}H-NMR: (400 MHz, $CDCl_3$) δ = 5.35 (m, 2H), 4.05 (t, *J* = 6.8 Hz, 2H), 2.04 (s, 3H), 2.01 (m, 4H), 1.61 (p, *J* = 7.0 Hz, 2H), 1.23-1.37 (m, 18H), 0.90 (t, *J* = 7.0 Hz, 3H) ppm; ^{13}C-NMR: (100 MHz, $CDCl_3$) δ = 171.2, 129.85, 64.6, 32.0, 29.7, 29.51, 29.49, 29.3, 29.2, 28.6, 27.2, 26.9, 25.9, 22.3, 21.0, 14.0 ppm.

Synthesis of (*Z*)-10-heneicosene:

Bromooctane (1.47 g, 7.6 mmol) and triphenylphosphine (2 g, 7.6 mmol) were dissolved in 40 ml Xylene and refluxed for 24h. The solvent was removed in vacuo and the residue was dissolved in 5 ml dichloromethane. Crude phosphonium salt was precipitated by slow dilution with n-hexane as a white solid, which was collected via filtration, washed thoroughly with n-hexane to remove unreacted starting compounds and dried in vacuo.
The Wittig-salt was dissolved in 20 ml anhydrous THF and cooled to -40°C, before sodium bis(trimethylsilyl)-amide (7.4 ml of a 1M solution in THF, 7.4 mmol) was added slowly. The mixture was allowed to warm to -20°C and stirred for 30 min before it was cooled to -40°C again. A solution of tridecanal (1.23 g, 6.2 mmol) in THF (5 ml) was added slowly and the mixture allowed warming to 20°C. After stirring for 1 h, the reaction mixture was diluted with hexane (25 ml), treated with water (30 ml) and saturated NH_4Cl-solution and extracted with hexane (3 x 25 ml). The combined organic phases were washed with brine and dried over Na_2SO_4. After filtration, the solvent was removed via rotavap and the residue was redissolved in hexane. The insoluble material was filtered off and washed with hexane. The filtrate was concentrated via rotavap and the residual oil was chromatographed on silica gel (25:1 hexane/EtOAc) to obtain methyl (*Z*)-10-heneicosene (1.15 g, 63% yield) as a colorless oil.
^{1}H-NMR: (400 MHz, $CDCl_3$) δ = 5.35 (m, 2H), 2.01 (q, *J* = 6.3 Hz, 4H), 1.22-1.37 (m, 30H), 0.88 (t, *J* = 6.9 Hz, 6H) ppm; 13C NMR: (100 MHz, CDCl3) δ = 129.9, 31.9, 31.90, 29.8, 29.7, 29.64, 29.61, 29.56, 29.4, 29.3, 29.2, 27.2, 22.7, 14.1 ppm

Synthesis of (*Z*)-9-Octadecen-1-yl acetate:

Oleyl alcohol (1 g, 3.7 mmol) was dissolved in CH_2Cl_2 (40 ml) and acetic anhydride (710 μl, 7.5 mmol) and triethylamine (1.05 ml, 7.5 mmol) were added, followed by 2-3 small crystals of DMAP. The mixture was stirred at room temperature and monitored via TLC. After consumption of the starting material (~3h) water was added (25 ml) and the mixture extracted with diethylether (3 x 25 ml). The combined organic phases were washed with water (20 ml) and brine (20 ml), dried over Na_2SO_4 and concentrated under vacuum. The residual oil was purified via column chromatography on silica gel (9:1 hexane/EtOAc) and (*Z*)-9-octadecen-1-yl acetate (1.05 g, 91 % yield) was obtained as a colorless oil.
^{1}H-NMR: (400 MHz, $CDCl_3$) δ = 5.35 (m, 2H), 4.05 (t, *J* = 6.8 Hz, 2H), 2.04 (s, 3H), 2.01 (q, *J* = 6.9 Hz, 4H), 1.61 (p, *J* = 6.9 Hz, 4H), 1.22-1.38 (m, 22H), 0.88 (t, *J* = 6.7 Hz, 3H) ppm; ^{13}C-NMR: (100 MHz, CDCl3) δ = 171.2, 130.0, 129.8, 64.6, 31.9, 29.8, 29.7, 29.5, 29.4, 29.3, 29.2, 29.18, 28.6, 27.2, 27.16, 22.7, 21.0, 14.1 ppm

Synthesis of *rac*-2-Tridecyl acetate:

2-Tridecanol (1 g, 5 mmol) was dissolved in CH_2Cl_2 (40 ml) and acetic anhydride (950 μl, 10 mmol) and triethylamine (1.4 ml, 10 mmol) were added, followed by 2-3 small crystals of DMAP. The mixture was stirred at room temperature and monitored via TLC. After consumption of the starting material (~4h) water was added (25 ml) and the mixture extracted with diethylether (3 x 25 ml). The combined organic phases were washed with water (20 ml) and brine (20 ml), dried over Na_2SO_4 and concentrated under vacuum. The residual oil was purified via column chromatography on silica gel (9:1 hexane/EtOAc) and *rac*-2-tridecyl acetate (1.01 g, 84 % yield) was obtained as a colorless oil.
^{1}H-NMR: (400 MHz, $CDCl_3$) δ = 4.88 (sext., J = 6.3 Hz, 1H), 2.02 (s, 3H), 1.22-1.65 (m, 20H), 1.20 (d, J = 6.3 Hz, 3H), 0.88 (t, J = 6.8 Hz, 3H) ppm; ^{13}C-NMR: (100 MHz, $CDCl_3$) δ = 170.7, 71.0, 35.9, 31.9, 29.62, 29.6, 29.55, 29.5, 29.4, 29.3, 25.4, 22.7, 21.3, 19.9, 14.1 ppm

Synthesis of R- *and* S-*2-Tridecyl acetate.*
The pure enantiomers of 2-Tridecyl acetate were separated using the same protocol used for the enzymatic separation of *R*- and *S*-(Z)-10-heptadecen-2-yl acetate.

Synthesis of *rac*-2-Pentadecyl acetate:

Synthesis of rac-*2-Pentadecanol.*
2-Pentadecanone (1,4 g, 6.2 mmol) was dissolved in ethanol (100 ml) and cooled to 0°C. $NaBH_4$ (780 mg, 20.6 mmol) was added in small portions and the mixture stirred for 1h at 0°C. Saturated NH_4Cl-solution (50 ml) and water (50 ml) was added carefully, before the mixture was extracted with diethylether (3 x 80 ml). The combined organic phases were washed with water (80 ml) and brine (80 ml), dried over Na_2SO_4 and concentrated under vacuum. The residual oil was purified via column chromatography on silica gel (3:1 hexane/EtOAc) and *rac*-2-pentadecanol (1.29 g, 91% yield) was obtained as a white wax.
^{1}H-NMR: (400 MHz, $CDCl_3$) δ = 3.78 (sext., J = 6.0 Hz, 1H), 1.21-1.51 (m, 24H), 1.18 (d, J = 6.0 Hz, 3H), 0.88 (t, J = 6.7 Hz, 3H) ppm; ^{13}C-NMR: (100 MHz, $CDCl_3$) δ = 68.2, 39.4, 31.9, 29.7, 29.65, 29.62, 29.0, 25.7, 23.5, 22.7, 14.1 ppm.

Synthesis of rac-*2-Pentadecyl acetate.*
rac-2-Pentadecanol (1.29 g, 5.6 mmol) was dissolved in CH_2Cl_2 (40 ml) and acetic anhydride (1.06 ml, 11 mmol) and triethylamine (1.5 ml, 11 mmol) were added, followed by 2-3 small crystals of DMAP. The mixture was stirred at room temperature and monitored via TLC. After consumption of the starting material (~4h) water was added (25 ml) and the mixture extracted with diethylether (3 x 25 ml). The combined organic phases were washed with water (30 ml) and brine (30 ml), dried over $MgSO_4$ and concentrated under vacuum. The residual oil was purified via column chromatography on silica gel (9:1 hexane/EtOAc) and *rac*-2-pentadecyl acetate (1.18 g, 78% yield) was obtained as a colorless oil.
^{1}H-NMR: (400 MHz, $CDCl_3$) δ = 4.89 (sext., J = 6.3 Hz, 1H), 2.02 (s, 3H), 1.43-1.64 (m, 2H), 1.22-1.375 (m, 22H), 1.20 (d, J = 6.3 Hz, 3H), 0.88 (t, J = 6.8 Hz, 3H) ppm; ^{13}C-NMR: (100 MHz, $CDCl_3$) δ = 170.7, 71.0, 35.9, 31.9, 29.7, 29.62, 29.6, 29.5, 29.4, 29.3, 25.4, 22.7, 21.3, 19.9, 14.1 ppm

Synthesis of R- *and* S-*2-Pentadecyl acetate.*
The pure enantiomers of 2-Pentadecyl acetate were separated using the same protocol used for the enzymatic separation of *R*- and *S*-(Z)-10-heptadecen-2-yl acetate.

Synthesis of Farnesyl acetate:

Farnesol (1 g, 4.5 mmol) was dissolved in CH_2Cl_2 (40 ml) and acetic anhydride (850 μl, 9 mmol) and triethylamine (1.25 ml, 9 mmol) were added, followed by 2-3 small crystals of DMAP. The mixture was stirred at room temperature and monitored via TLC. After consumption of the starting material (~3h) water was added (25 ml) and the mixture extracted with diethylether (3 x 25 ml). The combined organic phases were washed with water (30 ml) and brine (30 ml), dried over $MgSO_4$ and concentrated under vacuum. The residual oil was purified via column chromatography on silica gel (18:1 hexane/EtOAc) and farnesyl acetate (1.06 g, 89% yield) was obtained as a colorless oil.
^{1}H-NMR: (400 MHz, $CDCl_3$) δ = 5.34 (t, J = 7.1 Hz, 1H), 5.09 (m, 4H), 4.59 (d, J = 7.1 Hz, 2H), 1.94-2.16 (m, 11H), 1.71 (s, 3H), 1.68 (s, 3H), 1.60 (s, 6H) ppm; ^{13}C-NMR: (100 MHz, $CDCl_3$) δ = 171.1, 142.2, 135.4, 131.3, 124.3, 123.6, 118.3, 61.4, 39.7, 39.5, 26.7, 26.2, 25.7, 21.0, 17.7, 16.4, 16.0 ppm.

Synthesis of 2-Hexyl acetate:
2-Hexyl acetate was synthesized via acetylation of 2-hexanol using the protocol of Cen *et al.*[7]. The NMR-data are in accordance with those reported in literature.

Synthesis of *rac*-3-Pentadecyl acetate:

Synthesis of rac-*3-Pentadecanol.*
A solution of 1-bromododecane (4 g, 16 mmol) in anhydrous THF (10ml) was added dropwise to a stirred suspension of magnesium shavings (400 mg, 16.5 mmol) in anhydrous THF (30 ml). After the addition was complete, the reaction temperature was kept at 40° for 1h, before the gray suspension was allowed to settle. Via a syringe 35 ml of the supernatant solution were taken up and added slowly to a stirred solution of propanal (900 mg, 15.5 mmol) in anhydrous THF (40 ml) while keeping the temperature at 20°C. After 30 min saturated NH_4Cl-solution (20 ml) and water (20 ml) were added slowly and the mixture was extracted with diethylether (3 x 50 ml). The combined organic phases were washed with brine (50 ml), dried over $MgSO_4$ and concentrated under vacuum. Purification was done via column chromatography on silica gel (15:1 to 6:1 hexane/EtOAc) and *rac*-3-pentadecanol (2.56 g, 72% yield) was obtained as a white solid.
^{1}H-NMR: (400 MHz, $CDCl_3$) δ = 3.35 (m, 1H), 1.19-1.56 (m, 24H), 0.94 (t, J = 7.4 Hz, 3H), 0.88 (t, J = 6.8 Hz, 3H) ppm; ^{13}C-NMR: (100 MHz, $CDCl_3$) δ = 73.4, 37.0, 31.9, 30.1, 29.7, 29.73, 29.6, 29.4, 25.7, 22.7, 14.1 ppm.

Synthesis of rac-*3-Pentadecyl acetate.*
rac-3-Pentadecanol (1.52 g, 6.7 mmol) was dissolved in CH_2Cl_2 (60 ml) and acetic anhydride (1.26 ml, 13.3 mmol) and triethylamine (1.85 ml, 13.3 mmol) were added, followed by 2-3 small crystals of DMAP. The mixture was stirred at room temperature and monitored via TLC. After consumption of the starting material (~3h) water was added (40 ml) and the mixture extracted with diethylether (3 x 50 ml). The combined

organic phases were washed with water (50 ml) and brine (50 ml), dried over Na_2SO_4 and concentrated under vacuum. The residual oil was purified via column chromatography on silica gel (15:1 hexane/EtOAc) and *rac*-3-pentadecyl acetate (1.72 g, 95 % yield) was obtained as a colorless oil.

^{1}H-NMR: (400 MHz, $CDCl_3$) δ = 4.80 (p, *J* = 6.2 Hz, 1H), 2.04 (s, 3H), 1.53 (m, 4H), 1.20-1.35 (m, 20H), 0.88 (t, *J* = 7.3 Hz, 6H) ppm; ^{13}C-NMR: (100 MHz, $CDCl_3$) δ = 171.0, 75.6, 33.6, 31.9, 29.7, 29.64, 29.6, 29.5, 29.4, 26.9, 25.3, 22.7, 21.3, 14.1, 9.6 ppm.

Synthesis of (*Z*)-9-hexadecen-1-yl acetate:

Palmitoleyl alcohol (110 mg, 0.45 mmol) was dissolved in CH_2Cl_2 (4 ml) and acetic anhydride (85 μl, 0.9 mmol) and triethylamine (125 μl, 0.9 mmol) were added, followed by 1 small crystal of DMAP. The mixture was stirred at room temperature and monitored via TLC. After consumption of the starting material (~3h) water was added (5 ml) and the mixture extracted with diethylether (3 x 5 ml). The combined organic phases were washed with water (4 ml) and brine (4 ml), dried over Na_2SO_4 and concentrated under vacuum. The residual oil was purified via column chromatography on silica gel (9:1 *n*-hexane/EtOAc) and (*Z*)-9-hexadecen-1-yl acetate (115 mg, 90 % yield) was obtained as a colorless oil.

^{1}H-NMR: (400 MHz, $CDCl_3$) δ = 5.35 (m, 2H), 4.05 (t, *J* = 6.8 Hz, 2H), 2.04 (s, 3H), 2.01 (m, 4H), 1.62 (p, *J* = 6.9 Hz, 2H), 1.23-1.39 (m, 18H), 0.88 (t, *J* = 6.7 Hz, 3H) ppm; ^{13}C-NMR: (100 MHz, $CDCl_3$) δ = 171.2, 130.0, 129.8, 64.7, 31.8, 29.7, 29.4, 29.24, 29.2, 29.0, 28.6, 27.23, 27.18, 25.9, 22.7, 21.0, 14.1 ppm.

Synthesis of (*Z*)-11-Eicosen-1-yl acetate:

Synthesis of (Z)-Eicos-11-en-1-ol.

Methyl (*Z*)-eicos-11-enoate (1 g, 3.08 mmol) was dissolved in anhydrous diethylether (50ml) and cooled to 0°C. $LiAlH_4$ (234 mg, 6.16 mmol) was added and the mixture was allowed to warm to 20°C and stirred for 1 h. After cooling to 0°C, water (40 ml) and sat. NH_4Cl-solution (40 ml) was added dropwise. The mixture was extracted with *n*-hexane (3 x 50 ml), the combined organic phases were washed with brine (30 ml) and dried over $MgSO_4$. The solvent was removed in vacuum and the obtained material was dried. Further purification was not deemed necessary. (*Z*)-eicos-11-en-1-ol (910 mg, 99%) was obtained as a colorless wax.

^{1}H-NMR: (400 MHz, $CDCl_3$) δ = 5.35 (m, 2H), 3.63 (t, *J* = 6.7 Hz, 2H), 2.01 (q, *J* = 6.3 Hz, 4H), 1.56 (p, *J* = 7.0 Hz, 2H), 1.23-1.48 (m, 26H), 0.88 (t, *J* = 6.9 Hz, 3H) ppm; ^{13}C-NMR: (100 MHz, $CDCl_3$) δ = 129.92, 129.87, 63.1, 32.8, 31.9, 29.8, 29.6, 29.58, 29.55, 29.54, 29.4, 29.33, 29.31, 27.2, 25.8, 22.7, 14.1 ppm.

Synthesis of (Z)-11-Eicosen-1-yl acetate.

(*Z*)-Eicos-11-en-1-ol (910 mg, 3.06 mmol) was dissolved in CH_2Cl_2 (20 ml) and acetic anhydride (585 μl, 6.12mmol) and triethylamine (850 μl, 6.12 mmol) were added, followed by 2-3 small crystals of DMAP. The mixture was stirred at room temperature and monitored via TLC. After consumption of the starting material (~2h) water was added (35 ml) and the mixture extracted with *n*-hexane (3 x 40 ml). The combined organic phases were washed with water (50 ml) and brine (50 ml), dried over $MgSO_4$ and concentrated in vacuum. The residual oil was purified via column chromatography on silica gel (20:1 *n*-hexane/EtOAc) and (*Z*)-11-eicosen-1-yl acetate (940 mg, 91 % yield) was obtained as a colorless oil.

^{1}H-NMR: (400 MHz, $CDCl_3$) δ = 5.35 (m, 2H), 4.05 (t, J = 6.8 Hz, 2H), 2.04 (s, 3H), 2.01 (q, J = 6.3 Hz, 4H), 1.62 (p, J = 7.0 Hz, 2H), 1.20-1.38 (m, 26H), 0.88 (t, J = 6.9 Hz, 3H) ppm; ^{13}C-NMR: (100 MHz, $CDCl_3$) δ = 171.2, 129.92, 129.86, 64.7, 31.9, 29.8, 29.55, 29.53, 29.33, 29.3, 29.27, 28.6, 27.2, 25.9, 22.7, 21.0, 14.1 ppm.

Supplementary References

1 Pegel, M. Inferring the historical patterns of biological evolution. *Nature* **401**, 877–884. (1999).

2 Obbard, D. J. *et al.* Estimating divergence dates and substitution rates in the Drosophila phylogeny. *Mol Biol Evol* **29**, 3459-3473, doi:10.1093/molbev/mss150 (2012).

3 Russo, C. A. M., Mello, B., Frazão, A. & Voloch, C. M. Phylogenetic analysis and a time tree for a large drosophilid data set (Diptera: Drosophilidae). *Zoological Journal of the Linnean Society* **169**, 765-775, doi:https://doi.org/10.1111/zoj12062 (2013).

4 Prieto-Godino, L. L. *et al.* Functional integration of "undead" neurons in the olfactory system. *Sci Adv* **6**, eaaz7238, doi:10.1126/sciadv.aaz7238 (2020).

5 Markow, T. A. Evolution of *Drosophila* mating systems. *Evol Biol* **29**, 73–106 (1996).

6 Rao, A. V. R. & Reddy, D. R. A simple methodology for the synthesis of 3,4-Dihydroisocoumarins. *Synthetic Commun* **16**, 97-102, doi:Doi 10.1080/00397918608057694 (1986).

7 Cen, Y. X. *et al.* Highly focused library-based engineering of Candida antarctica lipase B with (S)-selectivity towards sec-alcohols. *Adv Synth Catal* **361**, 126-134, doi:10.1002/adsc.201800711 (2019).
