# Supplementary material for: Large-scale characterization of sex pheromone communication systems in Drosophila
Source: Nat Commun. 2021 Jul 6;12:4165. doi: 10.1038/s41467-021-24395-z (PMC8260797; doi:10.1038/s41467-021-24395-z)
Supplement: Supplementary file 3 — Reporting Summary [file 41467_2021_24395_MOESM3_ESM.pdf]

nature research

Corresponding author(s): Dr. Markus Knaden
Mohammed A. Khallaf

Last updated by author(s): 10.11.2020

Reporting Summary

Nature Research wishes to improve the reproducibility of the work that we publish. This form provides structure for consistency and transparency in reporting. For further information on Nature Research policies, see our Editorial Policies and the Editorial Policy Checklist.

Statistics

For all statistical analyses, confirm that the following items are present in the figure legend, table legend, main text, or Methods section.

n/a	Confirmed	
☐	☒	The exact sample size (*n*) for each experimental group/condition, given as a discrete number and unit of measurement
☒	☐	A statement on whether measurements were taken from distinct samples or whether the same sample was measured repeatedly
☐	☒	The statistical test(s) used AND whether they are one- or two-sided<br>*Only common tests should be described solely by name; describe more complex techniques in the Methods section.*
☒	☐	A description of all covariates tested
☐	☒	A description of any assumptions or corrections, such as tests of normality and adjustment for multiple comparisons
☐	☒	A full description of the statistical parameters including central tendency (e.g. means) or other basic estimates (e.g. regression coefficient) AND variation (e.g. standard deviation) or associated estimates of uncertainty (e.g. confidence intervals)
☐	☒	For null hypothesis testing, the test statistic (e.g. *F*, *t*, *r*) with confidence intervals, effect sizes, degrees of freedom and *P* value noted<br>*Give P values as exact values whenever suitable.*
☒	☐	For Bayesian analysis, information on the choice of priors and Markov chain Monte Carlo settings
☒	☐	For hierarchical and complex designs, identification of the appropriate level for tests and full reporting of outcomes
☐	☒	Estimates of effect sizes (e.g. Cohen's *d*, Pearson's *r*), indicating how they were calculated

Our web collection on statistics for biologists contains articles on many of the points above.

Software and code

Policy information about availability of computer code

Data collection	Gas-chromatography data were collected by MSD Chemstation software (F.01.03.2357); Electrophysiological data were collected and analyzed by Autospike software (version 3.7).
Data analysis	Gas-chromatography data were analyzed by XCMS online software (version 3.7.1); Statistical analyses were performed by GraphPad prism (version 8), and R (Version 1.1.447) ; Raw genetic reads were processed using Trimmomatic (version 0.32); Phylogenetic tree was processed by a branch-length stealing algorithm.

For manuscripts utilizing custom algorithms or software that are central to the research but not yet described in published literature, software must be made available to editors and reviewers. We strongly encourage code deposition in a community repository (e.g. GitHub). See the Nature Research guidelines for submitting code & software for further information.

Data

Policy information about availability of data

All manuscripts must include a data availability statement. This statement should provide the following information, where applicable:
- Accession codes, unique identifiers, or web links for publicly available datasets
- A list of figures that have associated raw data
- A description of any restrictions on data availability

All relevant data supporting the findings of this study and all unique biological materials generated in this study (raw data including genome sequences (66 species), movies for sexual behaviors of many drosophilids (1467 recordings for 55 Drosophila species), chemical profiles of virgin males, and virgin and mated females (over 1500 replicates), chemical syntheses of the sex-specific compounds, and other supplementary data) are available on accession code PRJNA669609 and https://dx.doi.org/10.17617/3.5w.

Field-specific reporting

Please select the one below that is the best fit for your research. If you are not sure, read the appropriate sections before making your selection.

☐ Life sciences ☐ Behavioural & social sciences ☒ Ecological, evolutionary & environmental sciences

For a reference copy of the document with all sections, see nature.com/documents/nr-reporting-summary-flat.pdf

Ecological, evolutionary & environmental sciences study design

All studies must disclose on these points even when the disclosure is negative.

Study description	Through a wide range series of whole genome sequencing, phylogenetic analyses, chemical identifications and syntheses, neuronal recordings, and behavioral experiments, we identified the olfactory sex pheromones of 99 species in the family Drosophilidae and demonstrate how these signals govern mate recognition and promote pre-mating isolation barriers.
Research sample	The study includes 99 Drosophila species (10-day-old males and females) that were selected to cover basically the full range along the phylogenetic tree of this genus. Flies were obtained from the National Drosophila Species Stock Centre and Kyoto stock center.
Sampling strategy	Based on our previously published data (Khallaf et al., 2020, Science Advances), chemical profiles of more than 5 replicates were analyzed per sex in all 99 species. For behavioral experiments, we collected data ranging from 24 to more than 100 replicates per species. In the SSRexperiments, the number of replicates is ranging from 3 and 10 per sensillum type in each species.
Data collection	Different people (Mohammed A. Khallaf: Chemical, behavioral, and electrophysiological experiments; Rongfeng Cui: evolutionary analyses; Jerrit Weißflog: chemical syntheses; Maide Erdogmus: electrophysiological analyses) were involved in the collection of data as stated in the Authors contribution section. All competition experiments were analyzed by double-blind observer (Ibrahim Alali).
Timing and spatial scale	All species are bred in the institute and data collection was performed within four years (from 2016 to 2020) prior to submission of the first manuscript.
Data exclusions	No data were excluded from the analysis.
Reproducibility	We did not repeat full experiments. We confirm that all attempts at replication were successful.
Randomization	No randomization was used, as it was not relevant to our experiments due to absence of allocation impact
Blinding	Double-blind data collection/analysis were done for the behavioral data. Chemical and electrophysiological experiments and evolutionary analyses could not blinded as they need direct observation.

Did the study involve field work? ☐ Yes ☒ No

Reporting for specific materials, systems and methods

We require information from authors about some types of materials, experimental systems and methods used in many studies. Here, indicate whether each material, system or method listed is relevant to your study. If you are not sure if a list item applies to your research, read the appropriate section before selecting a response.

Materials & experimental systems

n/a	Involved in the study	
☒	☐	Antibodies
☒	☐	Eukaryotic cell lines
☒	☐	Palaeontology and archaeology
☐	☒	Animals and other organisms
☒	☐	Human research participants
☒	☐	Clinical data
☒	☐	Dual use research of concern

Methods

n/a	Involved in the study	
☒	☐	ChIP-seq
☒	☐	Flow cytometry
☒	☐	MRI-based neuroimaging

Animals and other organisms

Policy information about studies involving animals; ARRIVE guidelines recommended for reporting animal research

Laboratory animals	We used 99 Drosophila species (10-day-old males and females) were all available from the National Drosophila Species Stock Centre and Kyoto stock center All names are included in the manuscript.
Wild animals	The study did not involve wild animals.

Field-collected samples	No field samples were included in this study.
Ethics oversight	No applicable for studies on Drosophila. Our study did not require ethical approval.

Note that full information on the approval of the study protocol must also be provided in the manuscript.
