# Supplementary material for: Large-scale characterization of sex pheromone communication systems in Drosophila
Source: Nat Commun. 2021 Jul 6;12:4165. doi: 10.1038/s41467-021-24395-z (PMC8260797; doi:10.1038/s41467-021-24395-z)
Supplement: Supplementary file 4 — Description of Additional Supplementary Files [file 41467_2021_24395_MOESM4_ESM.pdf]

Description of Additional Supplementary Files

File Name: Supplementary Data 1
Description: List of the 99 species, their classification according to NCBI, their stock number, and their breeding media. Whole-genome sequences (WGS) for 58 of theses 99 species (written in blue) are generated in this study, while the genomes of the other 41 species (written in green) were available from the corresponding reference.

File Name: Supplementary Data 2
Description: Pagel’s lambda estimates that explain the correlation between the chemicals among the different male and female species and their phylogenetic relationships. Lambda values are plotted in Supplementary Figure 1D; male-specific compounds are identified and shown in Supplementary Figure 2C.

File Name: Supplementary Data 3
Description: List of the names (according to IUPAC), appearance times, Kovat's Index, chemical formula, exact mass, and m/z of male and female-specific compounds.

File Name: Supplementary Data 4
Description: Names, chemical classes, vapor pressure at 25 °C, and the olfactory responses of the compounds used in the SSR experiments. 21 of these compounds are synthesized in this study, while the other 15 are commercially available through the listed vendors and CAS numbers.

File Name: Supplementary Data 5
Description: Species-species interaction network through at1 and at4 sensillum. The number of edges represent the olfactory interaction between species pairs, while the number of self-loops represent the intraspecific interactions. Correlation coefficient of each species is given and indicate the number of edges between the species divided by number of edges that could possibly exist.

File Name: Supplementary Data 6
Description: sheet1: Pagel’s lambda estimates that explain the phylogenetic corrected correlations between male-specific compounds and their neuronal response in at1 and at4 in all the tested 54 species. Sheet2: Pagel’s lambda estimates that explain the phylogenetic corrected correlations between male-specific compounds and their neuronal response in at1 and at4 in 16 pairs of closely related species.

File Name: Supplementary Data 7
Description: Values of sexual preferences of males and females of the different species. Statistical analysis between the tested groups is conducted via chisquare test.

File Name: Supplementary Data 8-16
Description: Phylogeny of Drosophila species within the groups of repleta, virilis, melanica, cardini, immigrans, melanogaster, obscura, willistoni, and saltans, respectively.
