# Supplementary material for: Large-scale characterization of sex pheromone communication systems in Drosophila
Source: Nat Commun. 2021 Jul 6;12:4165. doi: 10.1038/s41467-021-24395-z (PMC8260797; doi:10.1038/s41467-021-24395-z)
Supplement: Supplementary file 17 — Supplementary Data 13 [file 41467_2021_24395_MOESM17_ESM.rtf]

#NEXUSbegin trees;	tree tree_1 = [&R] (((((((((D.moj.sonorensis:0.001986,D.moj.baja:0.002039):4.78E-4,(D.moj.wrigleyi:0.003357,D.moj.mojavensis:0.003899):4.96E-4):0.003886,D.arizonae:0.006251):0.009534,D.navojoa:0.017031):0.002514,(D.wheeleri:0.014325,D.mulleri:0.012589):0.003466):0.016371,D.hamatofila:0.02316):0.005282,D.buzzatii:0.038882):0.011633,((D.mercatorum:0.031466,D.repleta:0.024331):0.004701,D.stalkeri:0.022912):0.015813):0.005758,(D.mettleri:0.047804,D.hydei:0.034368):0.006171):0.020784;end;
